# Novel Synergistic Synthesis of Ni-Doped Fe_3_O_4_ Nanocomposite: Adsorption Interactions with Heavy Metal Ions and Antimicrobial Studies of Hantzsch Products

**DOI:** 10.1021/acsomega.5c05934

**Published:** 2025-09-16

**Authors:** Ashutosh Gupta, Priyanshi Kamboj, Kaushik Pal, Jyoti Dagar, Aayushi Mandhan, Manvinder Kaur, Navneet Kaur, Harvinder Singh Sohal, George Z. Kyzas

**Affiliations:** † Department of Chemistry, 418665 Chandigarh University, Gharuan, Mohali, Punjab 140413, India; ‡ University Center for Research and Development (UCRD), Department of Physics, Chandigarh University, Ludhiana–Chandigarh State Hwy, Gharuan, Mohali, Punjab 140413, India; § Chitkara University Institute of Engineering and Technology, Chitkara University, Rajpura, Punjab 140401, India; ∥ Hephaestus Laboratory, School of Chemistry, Faculty of Sciences, 37791 Democritus University of Thrace, Kavala GR 65404, Greece; △ Department of Chemistry, Akal Degree College, Mastuana Sahib-148002, Sangrur, Punjab, India

## Abstract

This study explores the synthesis, catalytic performance, and dual functionality of Ni@Fe_3_O_4_ nanoparticles as both reusable catalysts and adsorbents for heavy metal ions. The Ni@Fe_3_O_4_ nanoparticles efficiently catalyzed the one-pot synthesis of 1,4-dihydropyridine (1,4-DHP) derivatives, achieving high yields (89%–96%) with excellent purity, as confirmed by advanced spectroscopic techniques. The synthesized 1,4-DHP derivatives, particularly those with polar groups at the para position (compounds **3d**, **3e**, and **3f**), exhibited significant antimicrobial activity comparable to that of standard pharmaceutical drugs. Additionally, the Ni@Fe_3_O_4_ nanoparticles exhibited high efficiency in adsorbing Pd­(II) and Cu­(II) ions, with optimal performance at a dosage of 0.07 g and pH 4, driven by enhanced protonation at acidic pH. The adsorption isotherm studies indicated a higher affinity for Pd­(II), reaching a maximum adsorption capacity of 968.18 mg/g, while Cu­(II) achieved 758.99 mg/g. These results underscore the versatility of Ni@Fe_3_O_4_ nanoparticles as promising multifunctional materials for applications in organic synthesis, antimicrobial treatments, and environmental remediation of heavy metals.

## Graphical abstract

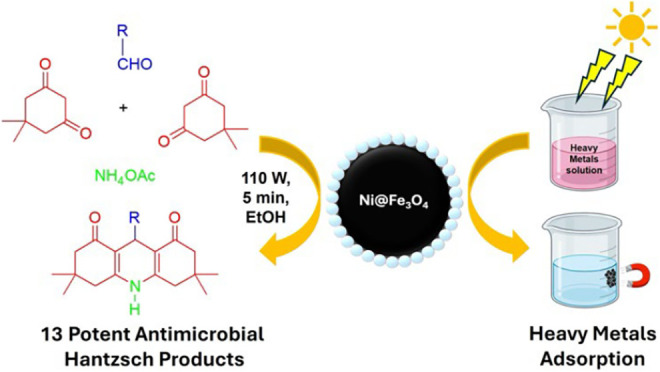

## 1 Introduction

Multicomponent reactions, sometimes called multicomponent assembly processes (MCAP), are increasingly becoming valuable methods of organic synthesis because they report the diversity and complexity of organic transformation.^1^ This involves the simultaneous reaction of three or more reactants to form a single product, making them highly efficient in costly organic synthesis.^2^ This process is suitable for the synthesis of structurally diverse organic compounds as well as for the synthesis of small drug-like materials such as nimodipine,^3^ amlodipine,^4^ and many more. In the production of bioactive derivatives of 1,4-dihydropyridine (1,4-DHP), multicomponent reactions have been extensively studied and utilized.

1,4-DHPs have diverse pharmacological profiles, and it is extensively studied for calcium channel blockers,^5^ antitumor,^6^ anti-inflammatory, antiallergic,^7^ antibacterial,^8^ and antioxidant agents.^9^ In the literature, several procedures have been described to produce 1,4-DHPs. It has been reported that a wide range of catalysts are studied to produce 1,4-DHP derivatives in multicomponent reactions. These catalysts include different Lewis acids, ZnCl_2_,^10^ (SnCl_4_),^11^ Bronsted acids Yb­(OTF)_3_,^12^ heterogeneous catalyst HClO_4_–SiO,^13^ zeolite,^14^ organocatalysts such as ceric ammonium nitrate (CAN),^15^ tertbutyl aluminum hydrogen sulfate,^16^ l-proline,^17^ nanocatalysts such as zinc oxide nanoparticles,^18^ iron oxide nanoparticles,^19^ Fe_3_O_4_ nanoparticles,^20^ carbon nanotubes,^21^ CuO nanoflakes^22^ have been employed. These catalysts have been utilized under various conditions and techniques, including stirring,^12^ conventional heating,^9^ refluxing,^10^ microwave irradiations,^4^ and ultrasound treatment.^12^ The use of other solvents like glycerol and water^17^ is a result of research’s current attention on the environmentally friendly elements of this reaction. In the synthesis of 1,4-DHP, some of the reactions are solvent-free^28^, while some reactions are completed by using solvents like ethanol,^23^ methanol,^24^ and so on.

Heavy metal adsorption using magnetic nano adsorbents offers a promising approach to efficiently remove toxic metals from aqueous solutions due to these nanoparticles’ unique magnetic and adsorption properties.^25^ Ni@Fe_3_O_4_ nanoparticles, composed of a nickel core and Fe_3_O_4_ (iron oxide) shell, possess a high surface area, magnetic properties, and strong attraction toward heavy metals, enabling efficient adsorption and easy magnetic separation from water.^26^ These nanoparticles facilitate the effective removal and detection of metals like lead, cadmium, and mercury by binding to metal ions through surface interactions, driven by electrostatic attraction, complexation, and ion exchange.^27,28^ The magnetic property enables easy recovery using an external magnet, simplifying both the adsorption and remediation process, thus positioning Ni@Fe_3_O_4_ nano adsorbents as a valuable tool in water purification and environmental monitoring.^29,30^

However, despite advances in catalysts for 1,4-DHP synthesis, challenges such as long reaction times, high catalyst loading, limited recyclability, and the use of harsh or toxic conditions persist in many reported methods. Recently, Ni@Fe_3_O_4_ nanoparticles have shown promise due to their magnetic recoverability, high surface area, and enhanced catalytic performance, but their application for microwave-assisted green synthesis of 1,4-DHP derivatives remains underexplored. Furthermore, combining catalytic activity with effective heavy metal adsorption in a single material presents an opportunity for multifunctional, sustainable approaches that have yet to be fully investigated.

Therefore, this study aims to develop and evaluate a reusable Ni@Fe_3_O_4_ nanocatalyst for rapid, efficient microwave-assisted synthesis of 1,4-DHP derivatives under mild and environmentally friendly conditions while also assessing its capacity as a magnetic nano adsorbent for heavy metal removal. This dual functionality underscores the potential of Ni@Fe_3_O_4_ nanoparticles for both synthetic organic chemistry and environmental remediation.

## 2 Experimental Section

### 2.1 Materials and Spectroscopic Investigations

All reagents and chemicals used in this study were sourced from Loba Chemie, Ludhiana, Punjab, India, and solvents were supplied by Chengshu Song Sheng Fine Chemical, Ludhiana, Punjab, India. Metal solutions were prepared fresh in deionized water to maintain the experimental consistency and purity standards.

The melting points of all synthesized Hantzsch products were determined by using a digital melting point apparatus (Avi Scientific India, Chandigarh University, Punjab, India). To confirm the functional groups and structural aspects of both the nanoparticles and the synthesized Hantzsch product, we used a PerkinElmer Spectrum II instrument from Chandigarh University, Punjab, India. NMR spectra were obtained using CDCl_3_ as solvent using a 500 MHz NMR spectrometer (Bruker Advance NEO, Punjab University, Chandigarh, India). The XRD patterns of the nanoparticles were collected at room temperature and exposed to monochromatic Cu–Kα radiation (λ = 1.5418 Å, 50 kV, 40 mA) over a 2θ range from less than 1° to greater than 150°, in 0.02° increments (Bruker D8 Advance, Chandigarh University, Punjab, India). Scanning electron microscopy images were captured in high vacuum mode with resolutions ranging from 30 nm (30 kV) to 15.0 nm (1.0 kV), with elemental analysis on microscopic sections of nanoparticles conducted by energy-dispersive X-ray spectroscopy (JSM IT500, Chandigarh University, Punjab, India). The magnetic properties of nanoparticles were investigated by using a series vibrating sample magnetometer (Lake Shore 7410, IIT, Roorkee, India). The zero-point charge and hydrodynamic dimensions of the particles were measured for nanoparticles (Zetasizer Nano-ZS90, Chandigarh University, Punjab, India). The metal ion concentrations adsorbed onto the synthesized Ni@Fe_3_O_4_ nano adsorbent were analyzed and quantified using Inductively Coupled Mass Spectroscopy (Agilent Technologies, Punjab Agricultural University, Ludhiana, Punjab, India).

### 2.2 Synthesis of Fe_3_O_4_ Nanoparticles

The magnetic nanoparticles were synthesized by dissolving 5.4 g of FeCl_3_.6H_2_O and 2.8 g of FeSO_4_.7H_2_O in 100 mL of deionized water under refluxing. To prevent the oxidation of Fe^2+^ ions, the solution was purged with nitrogen gas throughout the reaction. Subsequently, 10 mL of 25% aqueous ammonia (NH_4_OH) was added dropwise to the solution, maintaining the solution pH to 11, which promotes the formation of Fe_3_O_4_ nanoparticles. The reaction mixture was stirred for another 2 h at 80 °C, during which the nanoparticles gradually formed and the solution turned black. Once the reaction was completed, the nanoparticles were magnetically separated, washed thoroughly by deionized water and ethanol to remove unreacted ions and byproducts, and then dried at 60 °C for 2 h in a hot-air oven. The dried Fe_3_O_4_ nanoparticles were then ground into fine powder using a mortar and pestle and stored in an airtight vial for future use.

### 2.3 Synthesis of Ni@Fe_3_O_4_ Nanoparticles

To prepare Ni@Fe_3_O_4_ nanoparticles, 0.5 g of NiCl_2_.6H_2_O (2.1 mmol Ni) was dissolved in 50 mL of deionized water, and 1 M NaOH solution was gradually added until the pH reached 10. The solution was stirred for 2 h to precipitate nickel hydroxide. The precipitates were collected, washed with water and ethanol, and then dried in a vacuum oven. The dried nickel hydroxide was then reduced in a hydrogen atmosphere at 400 °C for 2 h to obtain nickel cores. To coat the nickel core with the Fe_3_O_4_ nanoparticle, 2.7 g of Fe_3_O_4_ (12.0 mmol Fe) nanoparticles were sonicated in 100 mL of deionized water. The nickel core was added to this solution, and the pH was adjusted to 10 using a 1 M NaOH solution while stirring. The mixture was stirred for additional 2 h to allow the formation of the Fe_3_O_4_ shell. The Ni@Fe_3_O_4_ magnetic nanoparticles were then collected using an external magnet, washed thoroughly twice with water and ethanol, and dried at 60 °C for 2 h in a hot air oven. After drying, the nanoparticles were ground into a fine powder with a mortar and pestle and stored in an airtight vial for later use. The molar ratio of Ni to Fe used in this synthesis was 1:6. This ratio was selected based on preliminary trials examining Ni:Fe ratios of 1:3, 1:6, and 1:9. The 1:6 ratio exhibited the best performance in catalytic and adsorption tests (data summarized in Supporting Information, Table S1), combining strong magnetic responsiveness with high surface activity and stability.

### 2.4 Synthesis of 1,4-DHP Derivatives Using Ni@Fe_3_O_4_ Nanoparticles

An aldehyde (5 mmol), dimedone (10 mmol), and ammonium acetate (10 mmol) were combined in a nonstoichiometric ratio in ethanol, as a solvent and using 5 mmol % Ni@Fe_3_O_4_ as a catalyst. The reaction was carried out in a microwave reactor at 110 W for 5 min, and progress was monitored by TLC using an ethyl acetate:*n*-hexane (7:3) solvent system, as shown in Scheme 1. After the reaction was complete, the product was kept settling down and the catalyst was further recovered by using a magnet, and the 1,4-DHP crystals were filtered and then washed using ethanol. The colorless crystals were collected and dried at 60 °C in an oven and later sent for structure elucidation.

**Scheme 1:**
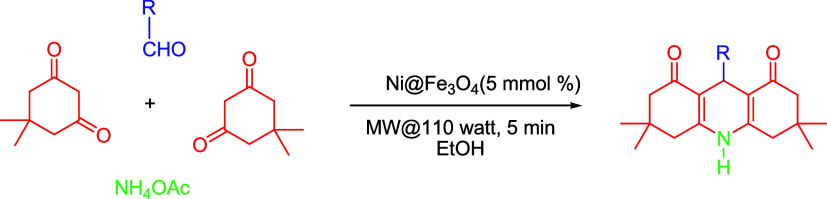
Synthesis of 1,4-DHP Derivatives **3a**–**m** Using Ni@Fe_3_O_4_ Nanoparticles

Table 1 highlights the synthesis of 1,4-DHP derivatives **3a–m** facilitated by Ni@Fe_3_O_4_ nanoparticles in ethanol, yielding highly pure compounds with significant efficiency, as evidenced by consistently high yields ranging from 89% to 96%. This outcome suggests that the Ni@Fe_3_O_4_ catalyst, paired with microwave irradiation, provides a robust and energy-efficient method for producing these derivatives. The *R*_f_ values, which measure the compounds’ relative mobility on a chromatographic medium, vary according to the substituents (R groups) on the phenyl ring, illustrating the influence of electron-withdrawing and electron-donating groups on polarity. For instance, derivatives with electron-withdrawing groups like −NO_2_, −Br, and −Cl tend to have lower *R*_f_ values, indicating increased polarity, while those with electron-donating groups such as −OH and −OMe show moderate R_f_ values due to their polarizing effect. Compared with literature values, the melting points (MP) of these compounds show close alignment, affirming their identity and purity. Slight deviations in melting points, as seen in derivatives like **3b** (3-Br C_6_H_4_), may be attributed to minor differences in experimental conditions but overall indicate the high reproducibility of the synthesis. Higher melting points in derivatives with substituents that facilitate intermolecular hydrogen bonding, such as −OH and −OMe, reflect greater stability in their crystalline forms. In contrast, lower melting points in compounds with bulky or flexible groups (e.g., vinyl or furyl groups) suggest reduced rigidity. This comprehensive analysis underscores the effectiveness of Ni@Fe_3_O_4_ nanoparticles in synthesizing a diverse range of structurally distinct 1,4-DHP derivatives with applications in antimicrobial studies and metal adsorption, supporting their potential as functional molecules in various chemical and biological applications. Slight melting point variations (*e.g*., **3b**: 303–304 °C vs. 305–307 °C) result from differences in purity, heating rate, or crystallization conditions. Such minor discrepancies are common and confirm that the compounds are consistent with reported structures.

**Table 1:** Synthesis of 1,4-DHP Derivatives **3a**–**m** Using Ni@Fe_3_O_4_ Nanoparticles in Ethanol

entry	R	*R*_f_	yield (%)	MP (°C)	Lit. MP (°C)	ref
**3a**	C_6_H_5_	0.85	96	280–281	279–280	^1^
**3b**	3-Br C_6_H_4_	0.77	95	303–304	305–307	^5^
**3c**	4-Br C_6_H_4_	0.83	96	239–241	238–240	^12^
**3d**	3-NO_2_ C_6_H_4_	0.75	96	275–276	273–275	^5^
**3e**	4-NO_2_ C_6_H_4_	0.69	95	280–281	282–283	^5^
**3f**	2-Cl C_6_H_4_	0.73	93	219–220	217–219	^5^
**3g**	4-Cl C_6_H_4_	0.81	94	227–228	228–229	^1^
**3h**	2-OH C_6_H_4_	0.82	95	307–308	305–306	^1^
**3i**	4-OMe C_6_H_4_	0.69	96	305–306	303–305	^1^
**3j**	4-Me C_6_H_4_	0.73	95	240–242	-	-
**3k**	4-OH, 3-OMe C_6_H_3_	0.78	91	232–234	-	-
**3l**	2-Furyl	0.75	91	230	-	-
**3m**	2-Thiophenyl	0.64	93	237–238	236–238	^3^

### 2.5 Antimicrobial Activities

The newly synthesized compounds, termed **3a**–**m**, were evaluated for antimicrobial activity against two Gram-positive bacterial strains, such as *Streptococcus pyogenes* MTCC 442 and *Bacillus subtilis* MTCC 441, three Gram-negative bacterial strains, such as *Staphylococcus aureus* MTCC 96, *Klebsiella pneumonia* MTCC 3384, and *Escherichia coli* MTCC 443, and three fungal strains, such as *Aspergillus niger* MTCC 281, *Aspergillus janus* MTCC 2751, and *Aspergillus sclerotium* MTCC 1008. Bacterial samples were cultured at 37 °C for 24 h and then preserved in nutrient broth, while the fungal strains were grown in malt extract medium at 28 °C for 72 h before inoculation. For bacterial assays, inocula were adjusted to approximately 1 × 10^6^ CFU/mL (0.5 McFarland standard) in the final test volume. Fungal spore suspensions were adjusted to 1 × 10^5^ spores/mL. A serial dilution method was used to test each compound in triplicate, with the chemicals dissolved in DMSO at concentrations of 2, 4, 8, 16, 32, 64, and 128 μg/mL. The final DMSO concentration in all assay wells did not exceed 1% (v/v), and a DMSO-only control at the same concentration was included to confirm no inhibitory effect on microbial growth.^8^

### 2.6 Adsorption Studies

In the adsorption experiments, 0.07 g of Ni@Fe_3_O_4_ nano adsorbent was mixed with each 10 mL of metal ion solution. The concentrations of the metal solutions were varied from 0.01 to 0.10 g/L at 303 K. The initial pH values were adjusted between 2.0 and 10.0 using 1 M HCl and 1 M NaOH as buffers. After shaking the solution for 2 h, the metal concentration was measured by using inductively coupled mass spectroscopy. The amount of metal ions adsorbed onto the surface of Ni@Fe_3_O_4_ nano adsorbent was calculated as the difference between the initial and final concentration at equilibrium. The experimental data was examined using nonlinear mathematical equations of Langmuir and Freundlich isotherm models expressed in eqs 1 and 2, respectively. For the Langmuir isotherm model

1 $$q_{e}=\frac{q^{0}bC_{e}}{1+bC_{e}}$$

where the maximal adsorption capacity is *q* ^0^ (mg/g), the equilibrium adsorption capacity is *q*_e_ (mg/g), the Langmuir constant is *b* (L/mg), and the equilibrium concentration of the metal ions is *C*_e_ (mg/L). For the Freundlich isotherm model

2 $$\log q_{e}=\log K_{F}+\frac{1}{n}\log C_{e}$$

where *n* is a parameter denoting adsorption strength and *K*_F_ (mg/g) is the Freundlich constant. All experiments were performed in triplicate in order to ensure the accuracy and reproducibility of the results.

## 3 Results and Discussion

### 3.1 Characterization of Ni@Fe_3_O_4_ Nanoparticles

#### 3.1.1 FTIR

The FT-IR spectra of Fe_3_O_4_ and Ni-doped Fe_3_O_4_ nanoparticles (Figure 1), providing insights into their molecular interactions and functional groups, reflecting both the nickel core and the iron oxide shell, which closely resembles that of pure Fe_3_O_4_.^31^ The FTIR spectrum of Fe_3_O_4_ nanoparticles exhibited a prominent peak around 546 cm^–1^, which was attributed to the Fe–O stretching vibration, confirming the formation of magnetite structure. A broad band at 3160 cm^–1^ corresponded to the O–H bending vibrations, indicating the presence of surface-adsorbed water molecules. Additionally, a peak observed near 1619 cm^–1^ was assigned to the stretching vibrations of the −OH group.

**Figure 1:**
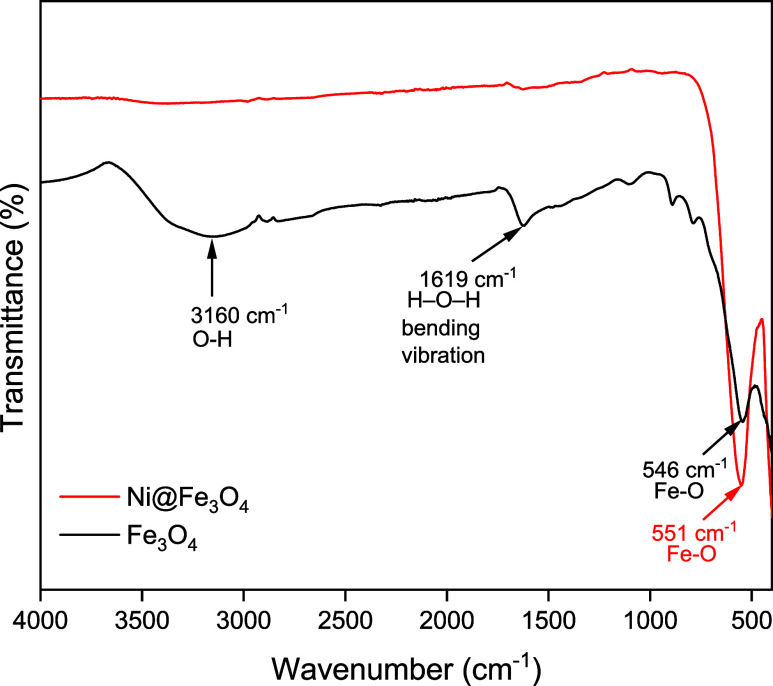
FT-IR spectra of the Fe_3_O_4_ and Ni@Fe_3_O_4_ nanoparticles.

Upon the incorporation of nickel into Fe_3_O_4_ to form Ni@Fe_3_O_4_ nanoparticles, slight shifts and intensity variations in IR bands were observed. The characteristic Fe–O vibration was evident at 551 cm^–1^, with the peak showing broadening and a minor shift. This broadening and minor shift is attributed to the incorporation of Ni atom on the surface or near the Fe–O lattice.^32^

#### 3.1.2 Ultraviolet (UV) Adsorption and Optical Energy Band Gap (E_g_) Analysis

The UV absorption spectra of the Fe_3_O_4_ and Ni@Fe_3_O_4_ nanoparticles, as shown in Figure 2, were analyzed to investigate their optical behavior. The Fe_3_O_4_ nanoparticles exhibited distinct absorption peaks at 206, 262, and 282 nm, with a broad absorption across the UV and visible regions, characteristic of Fe^2+^ to Fe^3+^ charge transfer transitions in the magnetite structure.^31^ After the incorporation of nickel, the Ni@Fe_3_O_4_ nanoparticles displayed additional peaks at 235, 297, and 474 nm, and showed an overall increase in absorption intensity, especially at lower wavelengths. Compared to pure Fe_3_O_4_, the Ni@Fe_3_O_4_ nanoparticles displayed a slightly higher and more sustained absorbance across the spectrum, indicating enhanced electronic interactions. A noticeable red shift in the absorption edge was observed, suggesting a reduction in the band gap due to Ni incorporation, while some peaks at lower wavelengths showed a slight blue shift, indicating changes in localized electronic states. The introduction of Ni atoms into the Fe_3_O_4_ matrix created additional localized states or modified the band structure, which contributed to the observed changes in the optical absorption.^32^ These results confirmed the successful incorporation of Ni and suggested that Ni doping enhanced the optical properties of the Fe_3_O_4_ nanoparticles by increasing the absorption at both UV and visible wavelengths.

**Figure 2:**
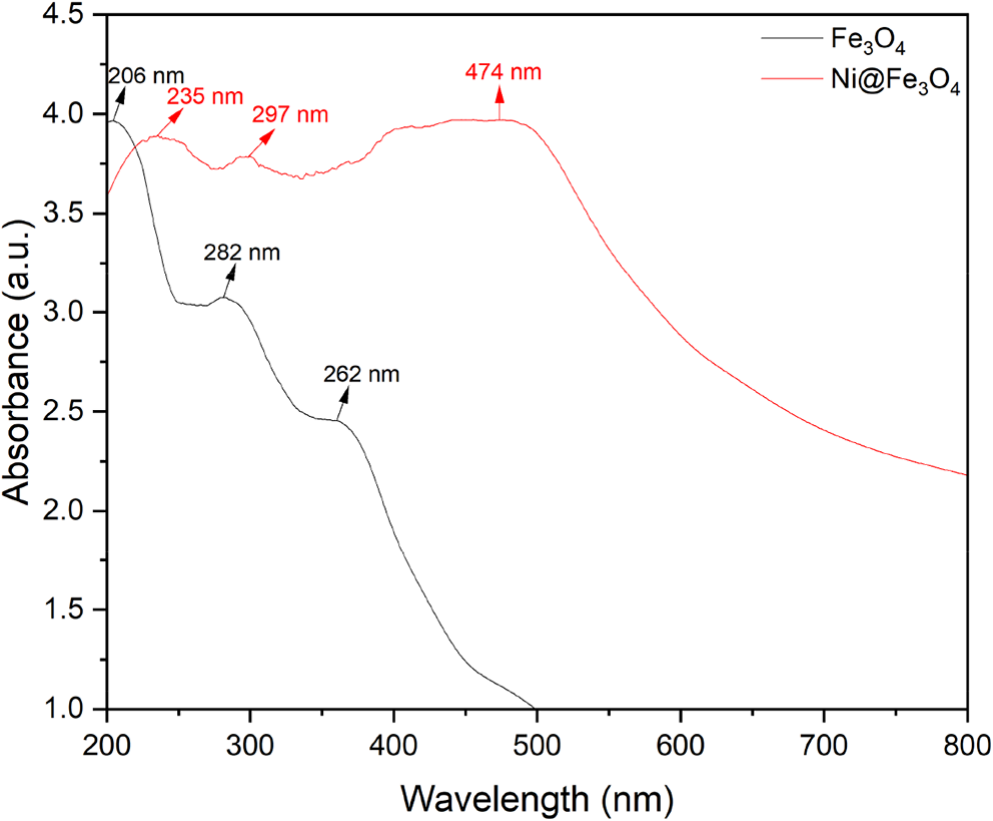
UV spectra of the Fe_3_O_4_ and Ni@Fe_3_O_4_ nanoparticles.

The Tauc plots for both Fe_3_O_4_ and Ni@Fe_3_O_4_ nanoparticles, as shown in Figure 3a,b, were constructed from UV absorption data to determine the optical band gap energies of the materials. The Fe_3_O_4_ nanoparticles exhibited a band gap of 4.54 eV, consistent with reported values for magnetite materials^31^ (Figure 3a). Upon the incorporation of Ni, the band gap of Ni@Fe_3_O_4_ nanoparticles decreased significantly to 2.31 eV^32^ (Figure 3b). This reduction in the band gap can be attributed to the introduction of Ni atoms into the Fe_3_O_4_ lattice, which modified the electronic structure by creating additional energy levels within the band gap or by altering the Fe–O bond interactions. The presence of Ni increased the density of defect states or facilitated charge delocalization, thereby reducing the energy required for electronic transitions. These observations suggest that Ni doping not only affected the optical absorption but also modified the electronic and structural properties of the Fe_3_O_4_ nanoparticles, making them more favorable for applications that require narrower band gap materials.

**Figure 3:**
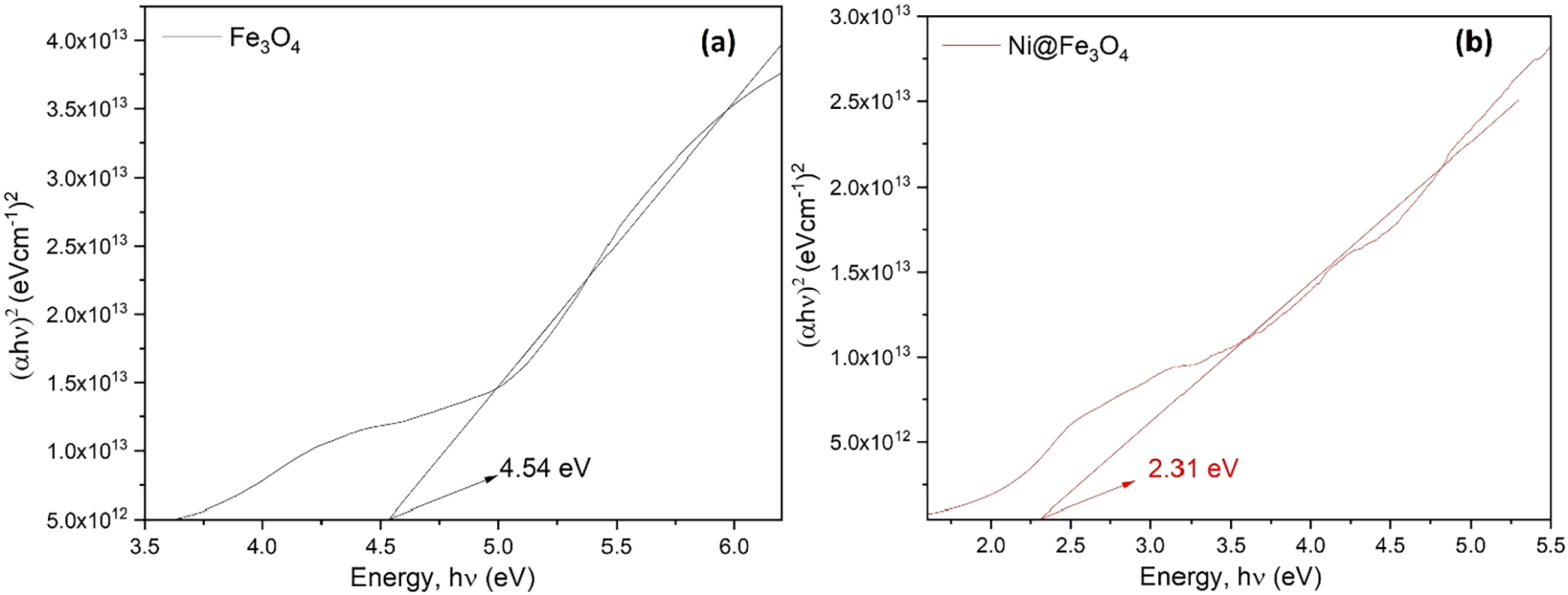
Tauc plots of (a) Fe_3_O_4_ and (b) Ni@Fe_3_O_4_ nanoparticles.

#### 3.1.3 DLS-Zeta Potential Analysis

The DLS and Zeta potential analysis of Fe_3_O_4_ and Ni@Fe_3_O_4_ nanoparticles are shown in Figure 4a,b. The DLS analysis determined the hydrodynamic size distribution, Figure 4a, while the zeta potential measurements assessed the surface charge and colloidal stability of the Fe_3_O_4_ and Ni@Fe_3_O_4_ nanoparticles in aqueous media. The DLS results showed that the pure Fe_3_O_4_ nanoparticles had an average hydrodynamic diameter of 50.83 nm, indicating a nanoscale particle size with good dispersion in the solution.^31^ However, upon Ni incorporation, the particle size slightly increases, with the Ni@Fe_3_O_4_ nanoparticles exhibiting a diameter of 68.75 nm. This increase is attributed to the surface modification caused by Ni^2+^ ions incorporated on the Fe_3_O_4_ lattice.

**Figure 4:**
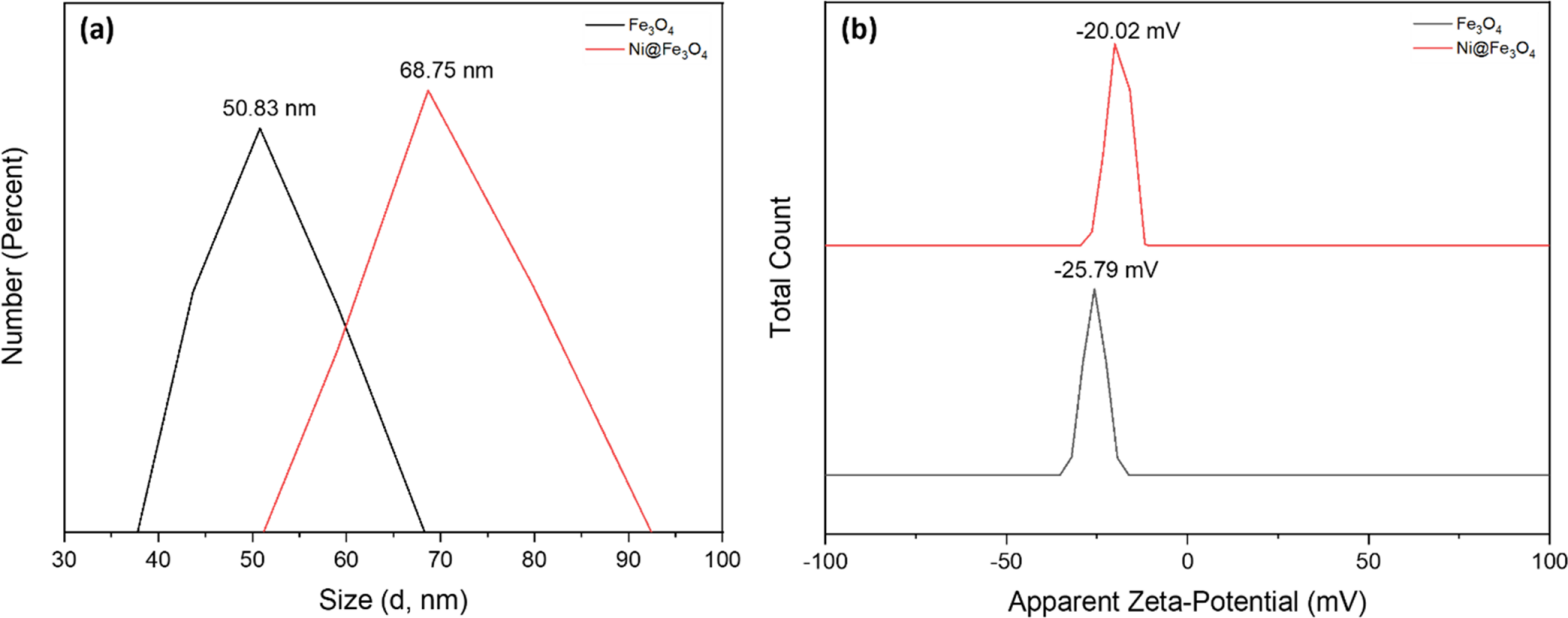
(a) DLS and (b) Zeta potential of Fe_3_O_4_ and Ni@Fe_3_O_4_ nanoparticles.

The zeta potential analysis indicated that pure Fe_3_O_4_ nanoparticles exhibited a −25.79 mV value, confirming excellent colloidal stability due to the strong electrostatic repulsion, as shown in Figure 4b. After Ni incorporation, Ni@Fe_3_O_4_ nanoparticles showed a slightly less negative zeta potential of −20.02 mV. This reduction in surface charge suggested changes in the surface chemistry, due to the substitution of Fe^3+^ ions by Ni^2+^ ions, which influenced the distribution of surface charges.^32^ Despite this reduction, the zeta potential of the Ni@Fe_3_O_4_ nanoparticles remains within the stable range, confirming their suitability for further applications in aqueous environments. These findings suggest that the nanoparticles retain their colloidal stability, which is essential for their practical use in various environmental and catalytic processes.

#### 3.1.4 SEM and EDS

The SEM images presented in Figure 5a–d provided crucial morphological insights into the surface structures and particle distributions of the Fe_3_O_4_ and nickel-doped Fe_3_O_4_ nanoparticles. In Figure 5a,b, Fe_3_O_4_ nanoparticles exhibited a dense and aggregated morphology with irregular spherical shapes. The particles appeared to be clustered together due to magnetic interactions and high surface energy, which promoted agglomeration. The surface appeared rough and consisted of uneven aggregates, which may have influenced the surface area and catalytic behavior of the nanoparticles.^31^

**Figure 5:**
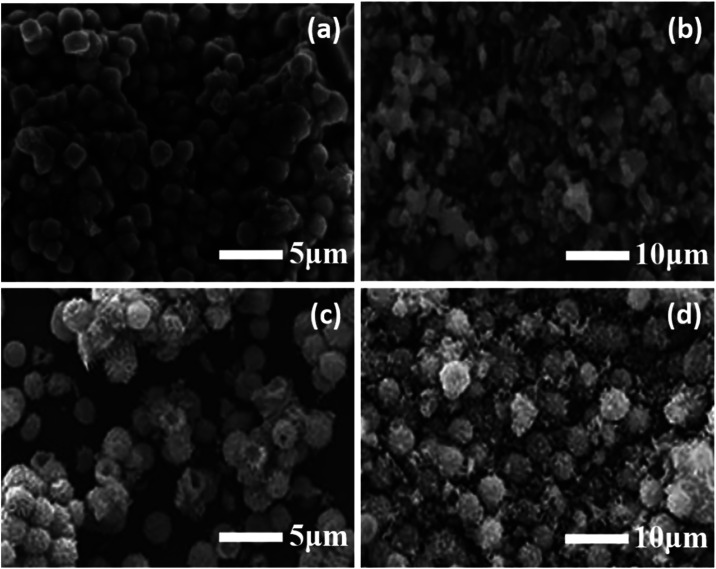
SEM images of (a, b) Fe_3_O_4_ and (c, d) Ni@Fe_3_O_4_ nanoparticles.

Upon modification with Ni, as observed in Figure 5c,d, a significant change in morphology was evident. The Ni@Fe_3_O_4_ nanoparticles appeared more well-dispersed and exhibited a more defined spherical structure with a relatively uniform size distribution. The surface texture appeared slightly rougher, suggesting the successful incorporation of Ni nanoparticles on the Fe_3_O_4_ surface. This morphological transformation was attributed to the stabilizing effect of Ni, which possibly reduced the magnetic interactions among Fe_3_O_4_ nanoparticles, thereby minimizing aggregation and improving the surface area. As a result, the SEM analysis confirmed the successful synthesis and morphological transformation of Fe_3_O_4_ nanoparticles upon Ni functionalization, highlighting improved structural uniformity in Ni@Fe_3_O_4_ nanoparticles.^32^

The EDS analysis shown in Figure 6a,b provided elemental composition data for both Fe_3_O_4_ and Ni@Fe_3_O_4_ nanoparticles, supporting the successful synthesis and surface modification of the sample. In Figure 6a, the EDS spectrum of Fe_3_O_4_ nanoparticles showed distinct peaks corresponding to iron (Fe) and oxygen (O), with no evidence of impurities, confirming the high purity of the Fe_3_O_4_ nanoparticles. The atomic percentages were calculated to be 62.47 ± 3.66% for oxygen and 37.53 ± 2.82% for iron, confirming the stoichiometry expected for Fe_3_O_4_. The consistent presence of Fe peaks at multiple energy levels, along with a strong O signal, supported the formation of Fe_3_O_4_ nanoparticles.

**Figure 6:**
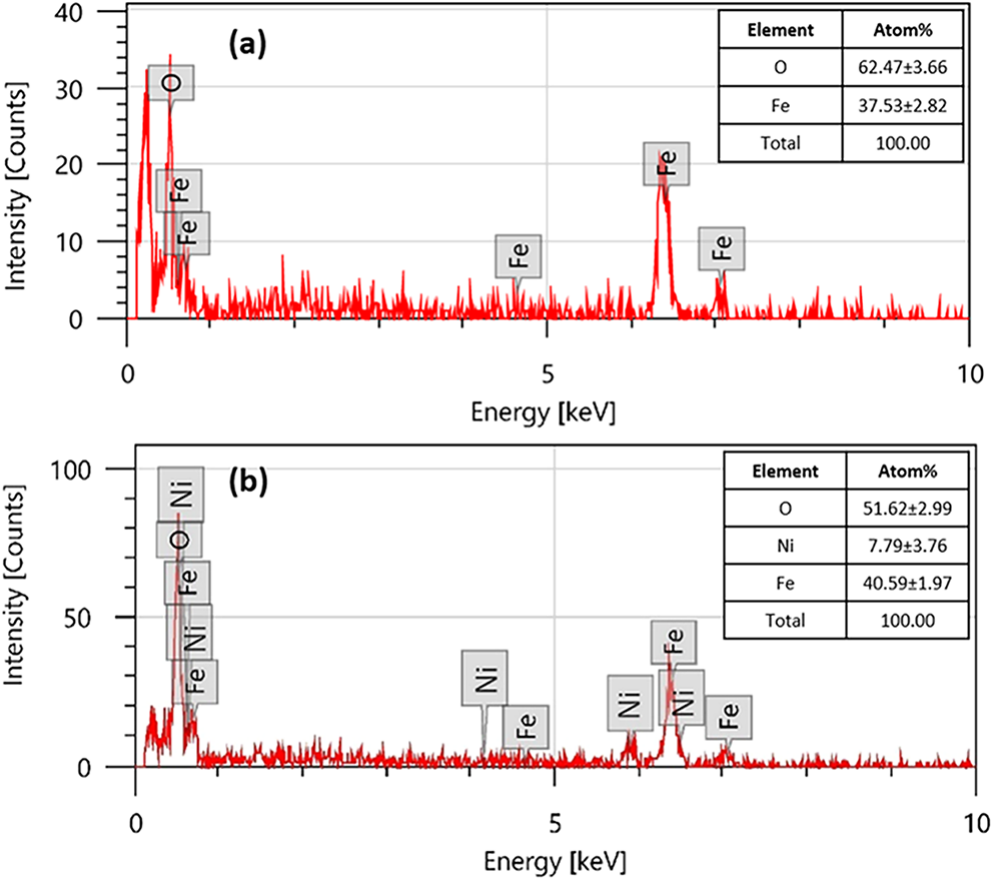
EDS spectra of (a) Fe_3_O_4_ and (b) Ni@Fe_3_O_4_ nanoparticles.

In contrast, Figure 6b displays the EDS results for Ni@Fe_3_O_4_ nanoparticles. Along with the characteristic peaks of Fe and O, several additional peaks corresponding to Ni were also observed. This confirmed the successful incorporation of Ni onto the Fe_3_O_4_ surface. The atomic composition revealed 51.62 ± 2.99% O, 7.79 ± 3.76 Ni, and 40.59 ± 1.97% Fe. The presence of Ni, even at a relatively moderate atomic percentage, was significant and demonstrated the successful surface functionalization of Fe_3_O_4_ nanoparticles with Ni. Further, the reduction in the O content compared to pure Fe_3_O_4_ nanoparticles was attributed to the surface coverage and incorporation of the Ni atom. This compositional modification agreed with SEM observations, where the morphological changes supported the presence and influence of Ni on the nanoparticle surface.

Figure 7 presents the histogram of the particle size distribution of Ni@Fe_3_O_4_ nanoparticles. The particle sizes were found to be within a broad range, predominantly between 20 and 40 nm, with an average size of around 29.48 nm. This confirmed the consistent nanoscale dimension of the synthesized catalyst, which is crucial for its high surface area and catalytic activity.

**Figure 7:**
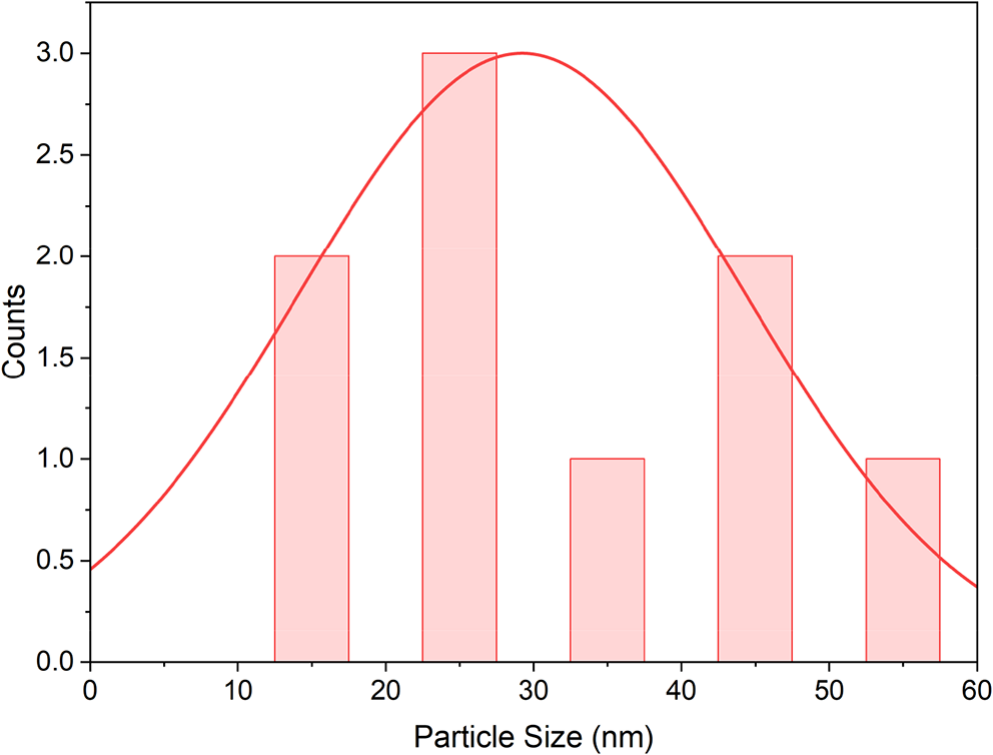
Histogram of the particle size distribution of Ni@Fe_3_O_4_ nanoparticles.

#### 3.1.5 TEM

In Figure 8a–d, the transmission electron microscopy (TEM) images provided significant insight into the particle size, morphology, and dispersion behavior of Fe_3_O_4_ and Ni@Fe_3_O_4_ nanoparticles. In Figure 8a,b, the Fe_3_O_4_ nanoparticles appeared as quasi-spherical clusters, indicating a relatively uniform size distribution with identical practical sizes of 25.22 nm. The dark contrast in the image suggested high electron density, typical of iron oxide, and the aggregation was attributed to magnetic interactions between the particles.^33^

**Figure 8:**
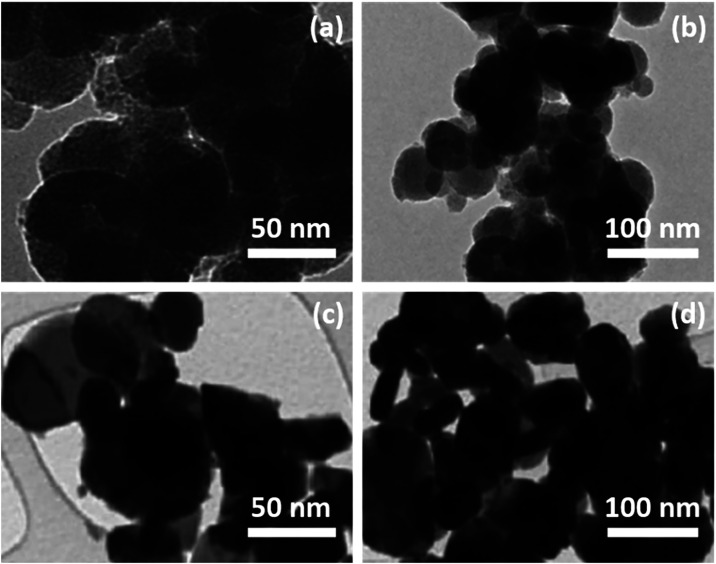
TEM images of (a, b) Fe_3_O_4_ and (c, d) Ni@Fe_3_O_4_ nanoparticles.

On the other hand, the Ni@Fe_3_O_4_ nanoparticles displayed in Figure 8c,d demonstrated a noticeable difference in contrast and shape. The presence of darker cores surrounded by a slightly shell structure supported the successful formation of a core–shell architecture. The core corresponded to the Fe_3_O_4_ nanoparticles, while the Ni deposition formed the outer shell. The particle size was still within the range, such as 29.48 nm, though some irregular morphologies suggest a heterogeneous coating. In Figure 8d, the aggregation appeared slightly more pronounced due to increased magnetic interactions from the bimetallic composition. Overall, the TEM analysis confirmed the successful synthesis of the Fe_3_O_4_ and Ni@Fe_3_O_4_ nanoparticles. The Ni modification did not drastically alter the particle size but did affect the morphology and aggregation behavior.^34^

#### 3.1.6 XRD

The XRD patterns of the Fe_3_O_4_ and Ni@Fe_3_O_4_ nanoparticles, measured in the range of 2θ = 0–100°, are shown in Figure 9 and provide critical insights into the crystalline nature and phase composition.^26^ The Fe_3_O_4_ nanoparticles exhibited characteristic diffraction peaks at 2θ values around 30.21°, 35.53°, 37.04°, 43.20°, 53.54°, 57.17°, and 62.86°, corresponded to (220), (311), (222), (400), (422), (511), and (440) planes, respectively, which match well with the standard JCPDS Card No. 19–0629. These sharp and intense peaks indicated high crystallinity and confirmed the successful synthesis of pure Fe_3_O_4_ nanoparticles. The calculated average crystalline size of Fe_3_O_4_ and Ni@Fe_3_O_4_ nanoparticles was found to be 30.22 and 31.13 nm, respectively, calculated using the Scherrer equation.^35^

**Figure 9:**
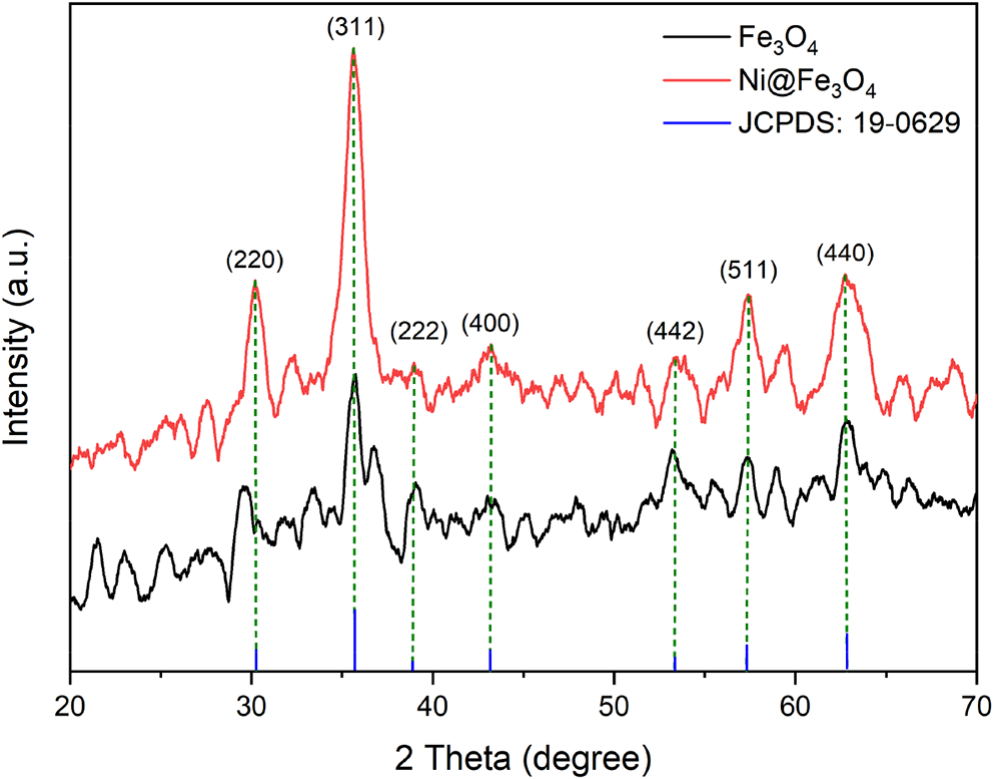
XRD spectra of the Fe_3_O_4_ and Ni@Fe_3_O_4_ nanoparticles.

After the incorporation of Ni, the XRD pattern of Ni@Fe_3_O_4_ nanoparticles retained the same characteristic peaks at 2θ values around 35.53°, 37.04°, 43.20°, 53.54°, 57.17°, and 62.86°, corresponded to (311), (222), (400), (422), (511), and (440) planes, respectively. However, a slight shift was observed at 30.21°, which corresponded to the (220) plane, indicating the successful incorporation of Ni into the Fe_3_O_4_ structure. The retention of primary Fe_3_O_4_ peaks alongside the new shift in diffraction pattern provided clear evidence of the structural modification caused by the Ni incorporation.^36^

#### 3.1.7 VSM

The magnetic properties of Fe_3_O_4_ and Ni@Fe_3_O_4_ nanoparticles exhibited significant changes upon the incorporation of Ni, which enhanced the magnetic performance of the Ni@Fe_3_O_4_ nanoparticles, as shown in Figure 10. For pure Fe_3_O_4_ nanoparticles, the saturation magnetization (*M*_s_) was 56.76 emu/g, indicative of their strong superparamagnetic nature, with strong coercivity (*H*_c_ = 85.5 Oe) and a high *M*_s_/*M*_r_ ratio of 22.7. This behavior suggested that the nanoparticles easily lose their magnetization once the external field was removed, which is characteristic of superparamagnetic materials.

**Figure 10:**
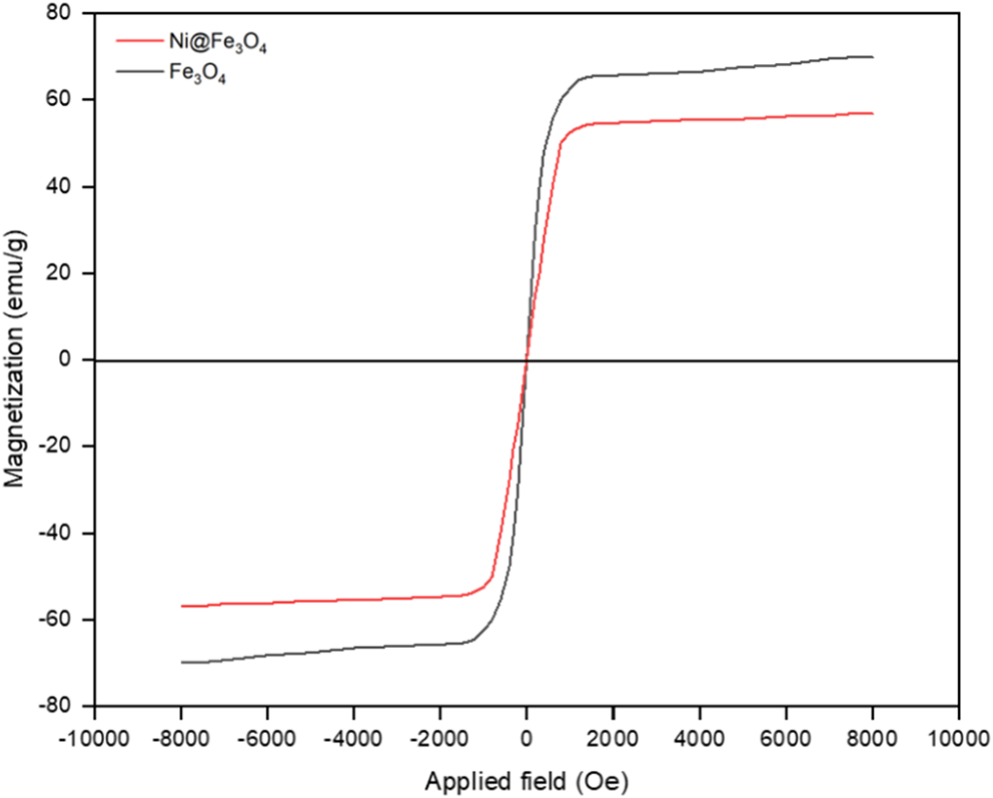
VSM spectra of the Fe_3_O_4_ and Ni@Fe_3_O_4_ nanoparticles.

However, upon incorporation of Ni into the Fe_3_O_4_ lattice, the saturation magnetization increased to 69.95 emu/g, reflecting the contribution of the ferromagnetic Ni^2+^ ions to the overall magnetization. Along with the increase in *M*_s_, the remanent magnetization (*M*_r_) also increased from 2.5 emu/g for pure Fe_3_O_4_ nanoparticles to 4.5 emu/g for Ni@Fe_3_O_4_ nanoparticles, indicating that the material retained more magnetization even after the external field is removed, suggesting a shift toward ferromagnetic behavior.^26^

Further, the coercivity increased to 112.4 Oe for Ni@Fe_3_O_4_ nanoparticles, which was a direct result of the ferromagnetic influence of Ni. This made the nanoparticles more resistant to demagnetization, thereby enhancing their stability in applications where magnetic recovery is essential. The increased coercivity also reflected the more stable magnetic state of Ni@Fe_3_O_4_ nanoparticles compared to the pure Fe_3_O_4_ nanoparticles.

Importantly, the observed magnetic enhancement cannot be attributed to Ni alone. The core–shell configuration likely introduced interfacial exchange coupling between the ferromagnetic Ni core and the superparamagnetic Fe_3_O_4_ shell. This interfacial interaction may facilitate spin alignment at the boundary, reduce spin disorder in the Fe_3_O_4_ layer, and thus enhance the overall magnetization. The decrease in *M*_s_/*M*_r_ ratio to 15.6 further supports the transition from a dominantly superparamagnetic to a mixed magnetic character with increased ferromagnetic contribution.^36^

Overall, the incorporation of Ni into the Fe_3_O_4_ lattice not only improved the magnetization properties but also shifted the magnetic behavior from superparamagnetic to more ferromagnetic, enhancing the potential of the material for various applications requiring high magnetic stability and efficient recovery.

### 3.2 Synthesis of 1,4-DHP Derivative (**3a**) Using Ni@Fe_3_O_4_ Nanoparticles

To optimize the synthesis conditions for 1,4-DHP derivative **3a**, we initially tested the influence of catalyst loading for Ni@Fe_3_O_4_ nanoparticles, as shown in Table 2. The experiment involved varying levels of catalytic loading of Ni@Fe_3_O_4_ nanoparticles, revealing that higher amounts of catalysts not only increased the yield but also reduced reaction times. However, once the saturation point was reached, only marginal increases in yield were observed with additional catalyst amounts. All reactions were carried out in triplicate to ensure reproducibility.

**Table 2:** Influence of Nanocatalyst’s Dosage on the Synthesis of Compound **3a**

catalyst dosage (mmol %)	time (min)	yield^a^ (%)
0	50	11
1	20	90
2	15	92
5	8	96
8	8	97
10	8	97

a Reaction efficiency with varying concentrations of Ni@Fe_3_O_4_ nanoparticles under MW irradiation at 100 W.

#### 3.2.1 Effect of Solvent

Multiple solvents were evaluated for the condensation reaction of benzaldehyde **1**, dimedone **2**, and NH_4_OAc **3**, including water, glycerol, ethanol, methanol, ethylene glycol, and polyethylene glycol (PEG) using 5 mmol % of Ni@Fe_3_O_4_ nanoparticles. Among the solvents tested, ethanol proved to be the most efficient, yielding the highest amount of the product, as shown in Table 3. The reaction times reported correspond to the times required to achieve the best yields for each solvent. This was attributed to the moderate polarity and excellent solvation properties of ethanol, which allowed better dispersion of the catalyst and enhanced interaction among the reactants and catalytic sites. In contrast, solvents with higher viscosity (such as glycerol or PEG) or lower polarity (such as water) led to reduced yields, due to limited molecular mobility or poor solubilization of components.

**Table 3:** Influence of Different Solvents on the Synthesis of Compound **3a** Using Ni@Fe_3_O_4_ Nanoparticles

solvent	time (mins)	yield^a^ (%)
Methanol	8	94
Glycerol	15	83
Water	18	29
Ethanol	8	96
Ethylene glycol	7	89
PEG	13	90

a Reaction efficiency with varying solvents using Ni@Fe_3_O_4_ nanoparticles (5 mmol %) under MW irradiation at 100 W.

#### 3.2.2 Effect of Power of Microwave Reactor

Examining the ideal power for a reaction conducted in an ethanolic catalytic solution reveals a compelling trend that raising the power substantially enhances the yield over time. However, increasing the power beyond the optimal point led to a decline in product yield due to the decomposition of the products at higher power, as presented in Table 4. The reaction times reported in Table 4 correspond to the durations required to achieve the best yields for each condition, reflecting the optimized times to ensure that the reactions proceeded to completion.

**Table 4:** Influence of Microwave Power on the Synthesis of Compound **3a**

watt	time (mins)	yield^a^ ^,^ ^b^ (%)
90	10	90
100	8	91
110	5	96
120	5	94
130	5	94

a Reaction conditions: Ni@Fe_3_O_4_ nanoparticles (5 mmol %) and ethanol (10 mL).

b Yield refers to the combined yield of all crops.

#### 3.2.3 Reusability of Ni@Fe_3_O_4_ Nanocatalyst

This study aimed to explore the reusability of magnetic Ni@Fe_3_O_4_ nanoparticles, given their easy recovery with an external magnet, as shown in Table 5. To test their reusability, we used them in a Hantzsch condensation reaction. After each reaction cycle, the nanoparticles were separated using an external magnet, thoroughly washed with distilled water and ethanol, and then dried. Before reuse, the catalyst was regenerated by thermal treatment at 600 °C for 1 h to restore its activity. This process allowed us to evaluate its effectiveness over multiple uses.

**Table 5:** Reusability of the Ni@Fe_3_O_4_ Nanocatalyst in the Synthesis of Compound **3a**

cycle	yield^a^ (%)
1	96
2	94
3	93
4	92
5	91

a Reusability of nanoparticles in the Hantzsch condensation reaction.

The catalyst exhibited excellent stability, with only a slight decrease in yield from 96% in the first cycle to 91% after the fifth cycle, indicating a minimal loss of catalytic activity. To further confirm the catalyst’s durability, IR and XRD analyses were performed on the fresh catalyst and again after the fifth cycle (Figure S14). The results showed minor or no significant changes in the characteristic peaks and structural features of the catalyst, demonstrating that the Ni@Fe3O4 nanocatalyst retained its chemical integrity and crystallinity throughout the reuse process. These findings collectively suggest that the catalyst was highly stable and reusable without considerable degradation.

#### 3.2.4 Comparison of Ni@Fe_3_O_4_ with Other Reported Catalysts for Hantzsch Condensation Reaction

The catalytic efficiency of Ni@Fe_3_O_4_ nanoparticles was compared with several reported nanocatalysts used for Hantzsch condensation reaction (Table 6). Unlike other catalysts such as ZnO, CuO nanoflakes, and NiO-ZrO_2_, which required longer reaction times (ranging from 20 to 140 min) and higher temperatures or reflux conditions, Ni@Fe_3_O_4_ demonstrated remarkable activity under mild conditions, completing the reaction in just 5 min under microwave irradiation with excellent yields (94–96%).

**Table 6:** Comparison of the Ni@Fe_3_O_4_ Nanocatalyst with Other Reported Nanocatalysts for the Hantzsch Condensation Reaction

catalyst	catalyst loading (mg)	solvent	reaction conditions	yield (%)	references
ZnO	10	solvent-free	40 min/110 °C	61–94	^37^
Cu-ZnO	20	water	reflux	89–90	^38^
CuO	15	EtOH:H_2_O	140 min/70 °C	80–90	^39^
NiO-ZrO_2_	15	EtOH	20–45 min/70 °C	89–98	^40^
Fe_3_O_4_	16	solvent-free	reflux	84–96	^41^
Ni@Fe_3_O_4_	12.5	EtOH	MW 5 min	94–96	this study

Even at a low catalyst loading of 12.5 mg, it outperformed or matched the efficiency of other systems, some of which required higher loadings and harsher conditions. This superior performance was attributed to the synergistic effect of Ni and Fe_3_O_4_, enhanced surface area, and magnetic recoverability of the catalyst, making it a highly efficient, green, and reusable option for Hantzsch synthesis.

### 3.3 Characterization of 1,4-DHP Derivative (3a)

A variety of spectroscopic techniques, including FT-IR, ^1^H, and ^13^C NMR, were employed to elucidate the structure of 1,4-DHP derivatives. The IR spectrum of compound **3a** in the series showed four notable absorptions at 3402 cm^–1^ (N–H), 2956.8 cm^–1^ (sp^2^, Ar C–H), 2877 cm^–1^, 2831 cm^–1^ (sp^3^, C–H), and 1582.19 cm^–1^ (C=O). In the ^1^H NMR spectrum (500 MHz, CDCl_3_) of molecule **3a**, δ, ppm: 11.89 (s, 1H, NH), 7.24–7.27 (d, 2H, Ar–H), 7.10–7.09 (d, 2H, Ar–H), 7.17–7.11 (s, 1H, CH), 2.47–2.22 (m, 8H, CH_2_), 1.22–1.67 (s, 6H, CH_3_), 1.09–1.06 (s, 6H, CH_3_). In the ^13^C NMR spectrum (125 MHz, CDCl_3_) of molecule **3a**, δ, ppm: 195.4, 191.6, 188.2, 46.3, 46.1, 31.4, 30.3, 28.4, 27.6, 27.1 corresponding to the primary 1,4-DHP structure, while aromatic region–containing carbon atoms were observed at 136.3, 130.9, 127.2, 118.1, and 114.2. Signals at 77.1, 77.0, and 75.3 nm correspond to the CDCl_3_ solvent. The spectral data for compound **3a** provide strong support for the proposed structure.

### 3.4 Possible Synthetic Mechanism

Figure 11 provides a proposed mechanistic route for the synthesis of 1,4-DHP derivatives **3a**–**m** catalyzed by Ni@Fe_3_O_4_ nanocatalyst. The proposed mechanism suggests that the initial activation of aldehyde’s carbonyl group makes it easier for the enol form of dimedone to be added nucleophilically to the carbonyl group to generate an intermediate with the removal of a water molecule, which is caused by the Ni@Fe_3_O_4_ nanocatalyst.^42^ The role of Ni and Fe centers in facilitating carbonyl activation and subsequent nucleophilic additions is supported by previous studies on Ni- and Fe-based catalytic systems, where similar coordination and activation steps have been confirmed through spectroscopic evidence such as in situ FTIR and NMR studies.^43^ The proton of NH_4_OAc can simultaneously activate the carbonyl group of dimedone, allowing for the subsequent nucleophilic addition of NH_3_ to its carbonyl group to generate an enamine intermediate and removal of the second water molecule. Then, by Michael’s addition of enamine to the activated intermediate and ring closure step with the removal of the third water molecule, the required products are formed.^42^ Water molecules are mainly byproducts of this reaction.

**Figure 11:**
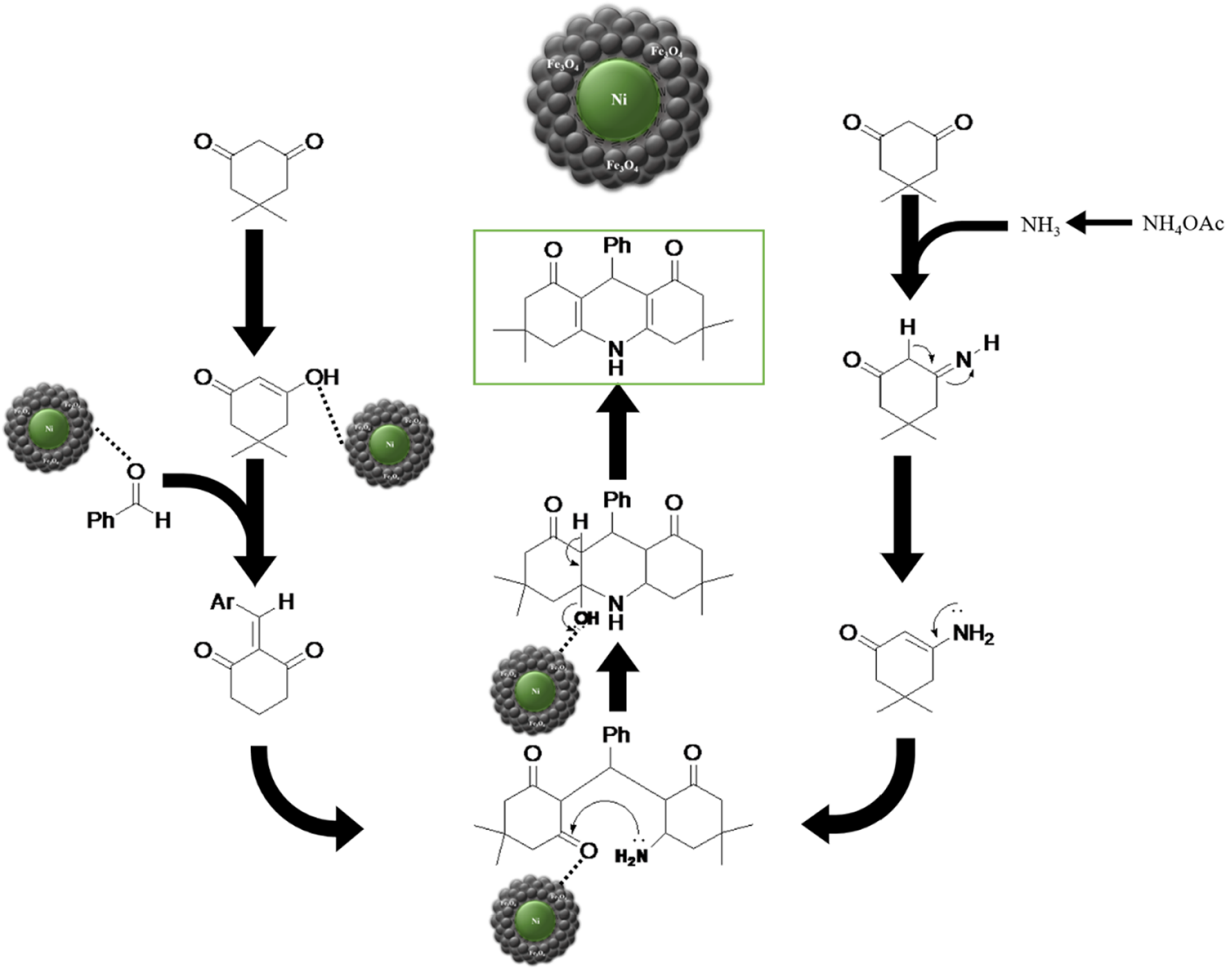
Synthesis mechanism of 1,4-DHP derivatives **3a**–**m** using Ni@Fe_3_O_4_ nanoparticles.

### 3.5 Evaluation of Antimicrobial Activity

The antimicrobial properties of the synthesized compounds **3a–m** were assessed using the minimum inhibitory concentration (MIC) method. The outcomes were contrasted to the reference medications such as Fluconazole and Amoxicillin, which were tested at 4 and 2 μg/mL, respectively. As shown in Table 7, the series **3a**–**m** demonstrated that a few of them have comparable activity to standard drugs. Compounds bearing polar groups at the para position of the phenyl ring, such as −OH (**3h** and **3k**) and –NO_2_ (**3d** and **3e**), demonstrated notable effectiveness, due to their ability to form hydrogen bonds with microbial proteins,^8^ exhibiting efficacy comparable to the standard drugs Amoxicillin and Fluconazole, with MIC values of 4 and 2 μg/mL, respectively. Blank (solvent-only) controls were also tested under identical conditions and showed no inhibition (MIC > 128 μg/mL) against all tested microorganisms, confirming that the observed antimicrobial effects were due solely to the synthesized compounds.

**Table 7:** MIC (μg/mL) of Synthesized 1,4-DHP Derivatives **3a**–**m** against Different Microbial Agents

	gram (+ve) bacteria		gram (-ve) bacteria			fungi		
compound	*B. Subtilis*	*S. Pyogenes*	*E. coli*	*K. Pneumonia*	*S. Aureus*	*A. Janus*	*A. Niger*	*A. Sclerotiorum*
blank	>128	>128	>128	>128	>128	>128	>128	>128
3a	32	8	8	8	32	8	32	8
3b	16	32	8	32	16	32	8	16
3c	32	16	16	16	32	16	8	16
3d	8	4	8	4	8	32	8	32
3e	4	4	4	8	4	4	4	8
3f	8	8	32	8	16	16	32	8
3g	32	64	8	8	32	8	64	128
3h	8	16	16	32	16	16	16	16
3i	8	8	32	8	16	16	32	8
3j	32	64	8	8	32	8	64	128
3k	8	16	4	8	8	8	8	8
3l	16	32	8	128	16	128	16	32
3m	8	16	16	128	128	16	32	16
amoxicillin	4	4	4	4	4	–	–	–
fluconazole	–	–	–	–	–	2	2	2

### 3.6 Adsorption Interactions

The adsorption of Pd­(II), Cd­(II), Cu­(II), Co­(II), and Ni­(II) onto the Ni@Fe_3_O_4_ nano adsorbent is primarily driven by favorable electrostatic attractions between the positively charged metal ions and the negatively charged surface of the nano adsorbent. The adsorption efficiency is influenced by the ionic radius of the metal ions, with smaller ions typically diffusing more easily into the pores. Increasing the dose of Ni@Fe_3_O_4_ nano adsorbent leads to higher removal efficiency, as more active sites are available for the binding of metal ions. The solution pH plays a critical role as lower pH values lead to competition with hydrogen ions, while higher pH can result in metal hydroxide precipitates. The adsorption time affects the uptake rate, with equilibrium usually reached within 30 to 120 min. The adsorption mechanism involves physisorption and chemisorption, with surface complexation playing a key role. Additionally, Ni@Fe_3_O_4_ can be regenerated for multiple adsorption cycles, making it a highly efficient and reusable nano adsorbent for removing heavy metal ions from aqueous solutions.

#### 3.6.1 Effect of Ni@Fe_3_O_4_ Nano Adsorbent Dose

The purpose of the nano adsorbent dosage variation studies was to find the optimum dose of Ni@Fe_3_O_4_ nano adsorbent for the adsorption of Pd­(II), Cd­(II), Cu­(II), Co­(II), and Ni­(II) at a concentration of 0.02 g/L for a contact time of 60 min at a temperature of 303 K. In this experiment, the nano adsorbent concentrations varied from 0.01 to 0.10 g. In this experiment, the removal efficiency of metal ions improved as the nano adsorbent dose increased, due to the greater availability of active adsorption sites on the Ni@Fe_3_O_4_ nano adsorbent. At 0.07 g concentration, the adsorption efficiency reaches equilibrium, suggesting that the active adsorption sites are becoming saturated, while on further increase in nano adsorbent dose, no significant improvement was observed, as shown in Figure 12. Among these metal ions, Cu­(II) and Pd­(II) show the highest removal efficiencies of 91.3% and 88.1%, respectively, while Cd­(II), Co­(II), and Ni­(II) exhibit relatively lower efficiencies, such as 44.9, 49.9 and 48.9%, respectively. As a result, 0.07 g was determined to be the optimal amount of Ni@Fe_3_O_4_ nano adsorbent for further studies on the removal of Cu­(II) and Pd­(II), as these are the only metal ions with significant affinity for adsorption from aqueous solutions.

**Figure 12:**
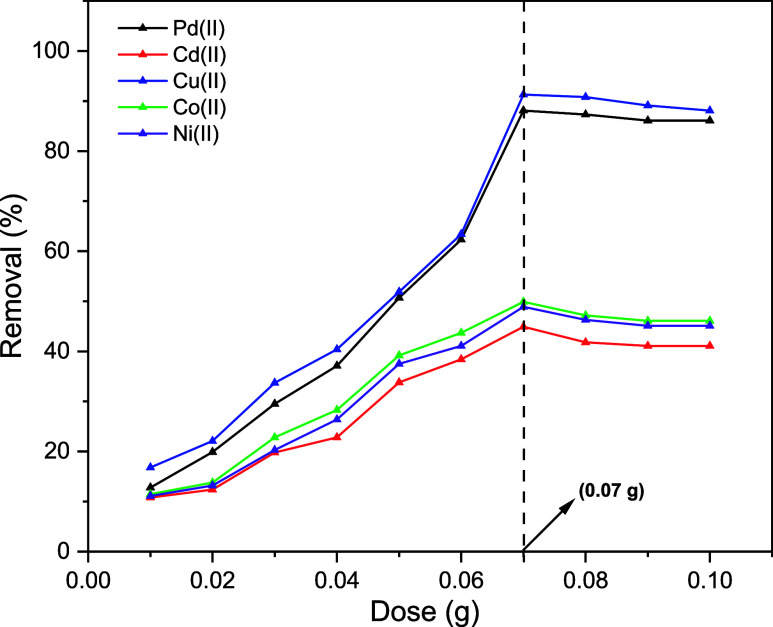
Dosage effect on the metal adsorption using Ni@Fe_3_O_4_ nano adsorbent.

#### 3.6.2 Influence of pH

In this experiment, the pH of the metal ion solution was studied using Ni@Fe_3_O_4_ nano adsorbent for the removal of Pd­(II) and Cu­(II). The pH was varied from 2.0 to 10.0, while keeping other parameters constant, including a nano adsorbent dose of 0.07 g in a 1000 mL solution at 303 K. With the help of 1 M HCl and 1 M NaOH, the pH of the mixture was adjusted. The effect of the pH on the adsorption behavior was assessed for Pd­(II) and Cu­(II), as shown in Figure 13a. Rising pH levels were shown to improve the adsorption capabilities of Pd­(II) and Cu­(II) on Ni@Fe_3_O_4_ nano adsorbent. For the metal ions Pd­(II) and Cu­(II), the maximum adsorption was seen at pH 4, 92.3 and 95.3%, respectively. Moreover, zeta potential analysis revealed that the Ni@Fe_3_O_4_ nano adsorbent had a zero-point charge of 6.3, as seen in Figure 13b. This suggests that the acidic conditions were favorable for metal ions adsorption on the nano adsorbent, and the removal efficiency was kept relatively high at pH 4. This is because the protonation of the nano adsorbent was increased at lower pH conditions, leading to higher adsorption of metal ions over the nano adsorbent surface. This observation was consistent with the previously reported study.^30^

**Figure 13:**
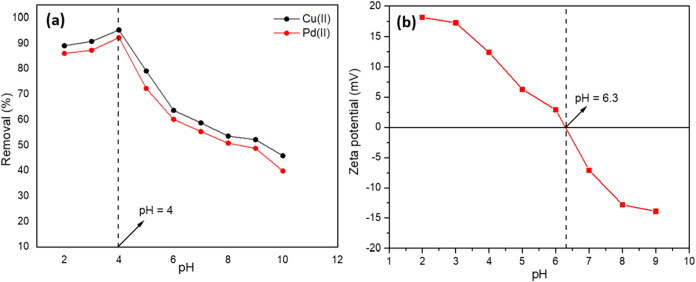
(a) Effect of pH on the removal efficiency of Pd­(II) and Cu­(II) using Ni@Fe_3_O_4_ nano adsorbent. (b) Determination of zero-point charge (pH_zpc_) of Ni@Fe_3_O_4_ nano adsorbent.

#### 3.6.3 Adsorption Isotherms

The experimental data were evaluated using the Langmuir and Freundlich isotherm models to understand the adsorption mechanisms of Pd­(II) and Cu­(II) ions on Ni@Fe_3_O_4_ nano adsorbent at 0.02 g/L concentration, as shown in Figure 14. The adsorption capacities for Pd­(II) and Cu­(II) reached 968.18 and 758.99 mg/g at an equilibrium concentration of 0.07 g/L, respectively. These results indicated that the adsorbent has a significantly higher affinity for Pd­(II) compared to Cu­(II), suggesting more efficient utilization of adsorption sites for Pd­(II). Additionally, Langmuir constant (*b*) is 0.00776 L/mg for Pd­(II) and 0.00358 L/mg for Cu­(II), suggesting that Pd­(II) reflects stronger interaction and greater adsorption onto the active sites of Ni@Fe_3_O_4_ nano adsorbent.

**Figure 14:**
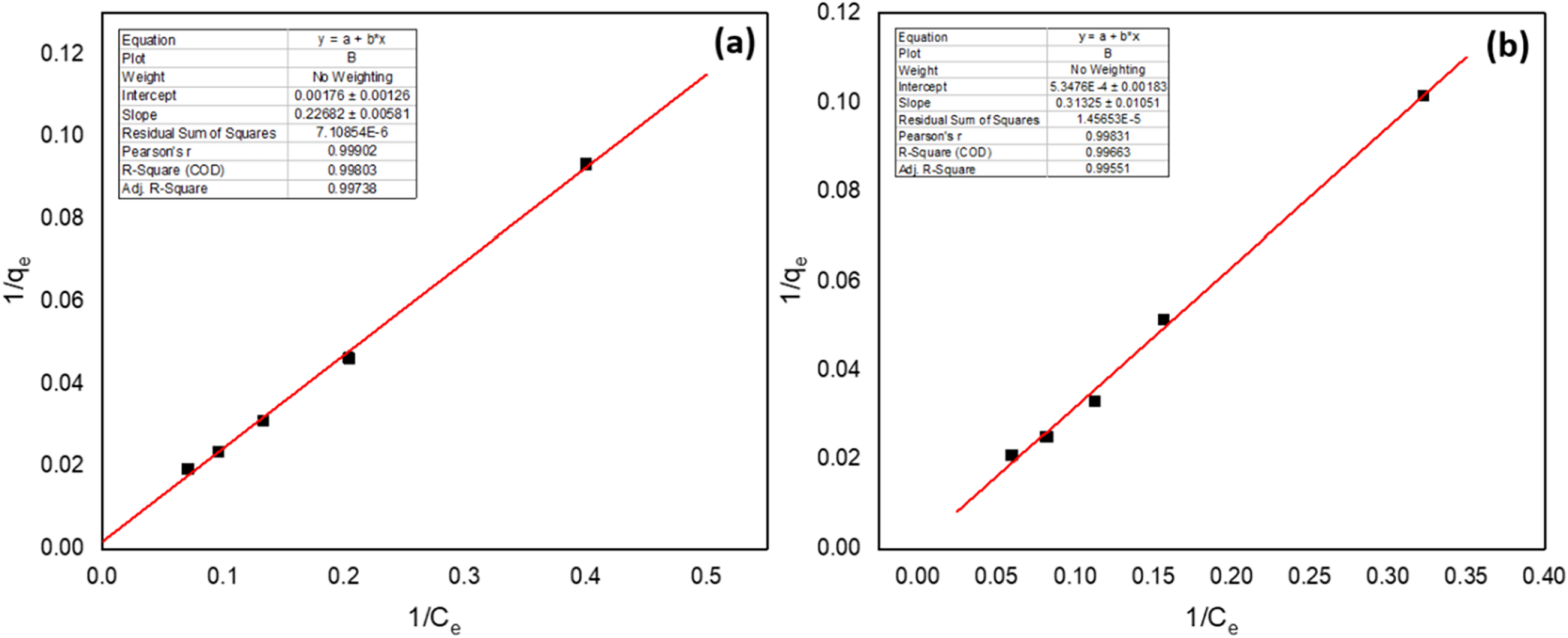
Langmuir isotherm plots of (a) Pd­(II) and (b) Cu­(II).

In contrast, the Freundlich isotherm model parameters show a different perspective of the adsorption characteristics, as shown in Figure 15. The Freundlich adsorption capacity, *K*_F_, for Pd­(II) is 4.84 mg/g, while that for Cu­(II) is 3.35 mg/g. These values further affirm the superior adsorption capacity of Ni@Fe_3_O_4_ nano adsorbent for Pd­(II). The Freundlich intensity factor (*n*) is calculated to be 3.09 and 2.38 for Pd­(II) and Cu­(II), respectively, suggesting that Pd­(II) demonstrates a higher intensity of adsorption compared to Cu­(II).

**Figure 15:**
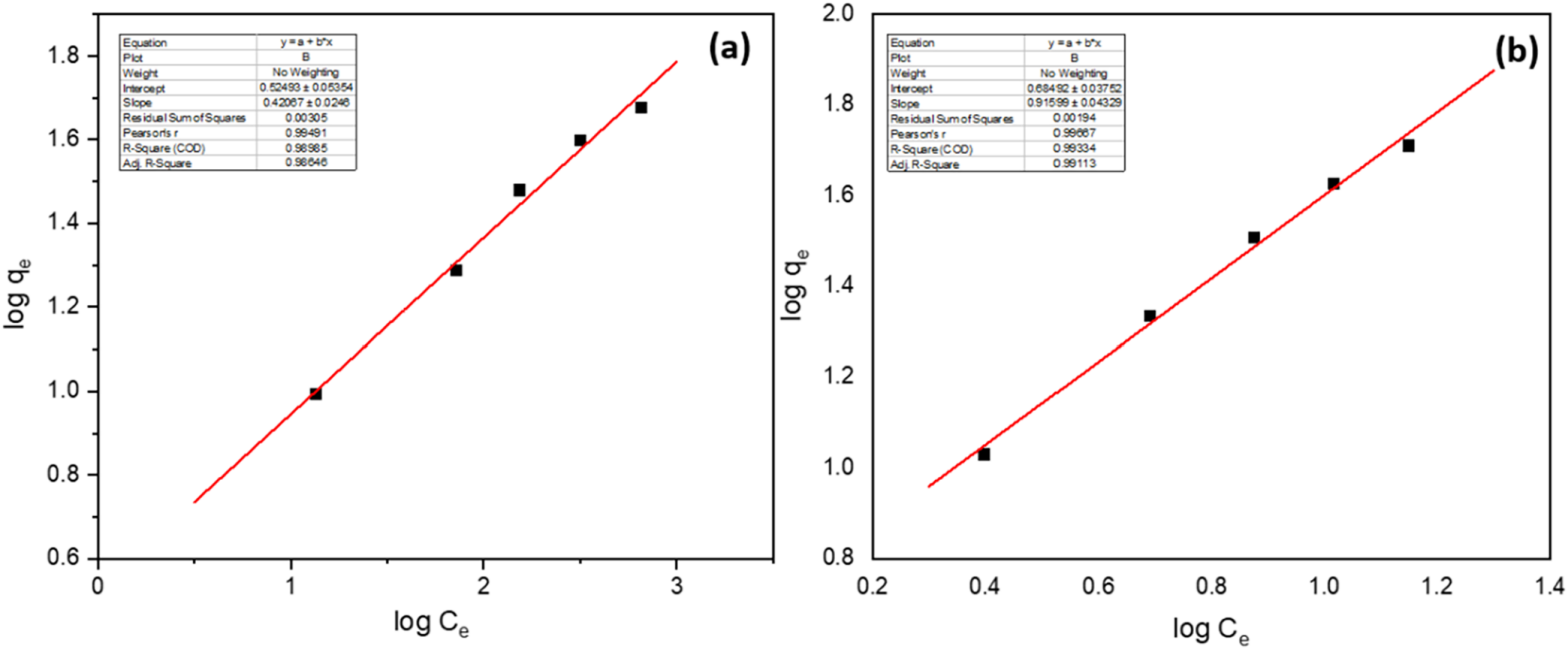
Freundlich isotherm plot of (a) Pd­(II) and (b) Cu­(II).

The comparative analysis of the adsorption isotherms for Pd­(II) and Cu­(II) highlights the adsorbent’s differential behavior with respect to the Pd­(II) and Cu­(II). The results indicate that the adsorbent exhibits a strong preference for Pd­(II), as evidenced by the higher values of *q* ^0^ and *K*_F_ and favorable *n* values in the Freundlich model. The regression coefficient values (*R* ^2^) for both models, which are close to one, as shown in Table 8, suggested that the adsorption process follows both isotherm models, indicating the complex nature of adsorption of Pd­(II) and Cu­(II) on the surface of Ni@Fe_3_O_4_ nano adsorbent. This dual fitting implied the involvement of both chemisorption, as described by the Langmuir model, indicating monolayer adsorption with stronger interactions, and physisorption, as described by the Freundlich model, representing multilayer adsorption on heterogeneous surfaces.

**Table 8:** Parameters of Langmuir and Freundlich Isotherm Models for Metal Ions Using the Ni@Fe_3_O_4_ Nano Adsorbent

	Langmuir isotherm model			Freundlich isotherm model		
heavy metal ion	*q* ^0^ (mg/g)	*b* (L/mg)	*R* ^2^	*K*_F_ (mg/g)	*n*	*R* ^2^
Pd(II)	868.18 ± 12.45	0.00776 ± 0.00022	0.99803 ± 0.00003	4.84 ± 0.15	3.09 ± 0.02	0.99334 ± 0.00001
Cu(II)	758.99 ± 15.38	0.00358 ± 0.00031	0.99663 ± 0.00005	3.35 ± 0.23	2.38 ± 0.05	0.98985 ± 0.00003

#### 3.6.4 Adsorption Comparisons

Among the nano adsorbents tested, including Fe_3_O_4_@ZIF-8, Fe_3_O_4_@SiO_2_-EDTA, and magnetic graphene oxide, the Ni@Fe_3_O_4_ nano adsorbent showed the highest adsorption capacities for both Pd­(II) (968.18 mg/g) and Cu­(II) (758.99 mg/g), showing a marked improvement over the reported nano adsorbents, as shown in Table 9. Ni@Fe_3_O_4_ exhibited higher adsorption capacity for Pd­(II) compared to Cu­(II), which was attributed to differences in their chemical properties and interactions with the catalyst surface. Pd­(II) has a larger ionic radius and higher polarizability than Cu­(II), allowing for stronger coordination with the surface Ni and Fe active sites. Additionally, Pd­(II)’s softer acid character leads to stronger complexation with the donor atoms on the Ni@Fe_3_O_4_ surface, enhancing its selective adsorption.

**Table 9:** Comparison of Ni@Fe_3_O_4_ Nano Adsorbent with Other Reported Nano Adsorbents for Adsorption Pd­(II) and Cu­(II)

nano adsorbent	adsorption capacity of Pd(II) (mg/g)	adsorption capacity of Cu(II) (mg/g)	references
Fe_3_O_4_@ZIF-8	719.42	301.33	^44^
Fe_3_O_4_@SiO_2_-EDTA	114.94	36.9	^45^
Magnetic graphene oxide	326.72	353.59	^46^
Ni@Fe_3_O_4_	968.18	758.99	this work

The superior adsorption performance of Ni@Fe_3_O_4_ is likely due to the synergistic effects between nickel and Fe_3_O_4_, which increase the number of active sites and enhance the surface properties for the metal ion adsorption. For instance, Fe_3_O_4_@ZIF-8, although it demonstrated relatively good performance with 719.42 mg/g for Pd­(II) and 301.33 mg/g for Cu­(II), still falls short compared to Ni@Fe_3_O_4_ Since the ZIF-8 framework is known for its high surface area which enhances the metal ion attraction, the combination of Ni and Fe_3_O_4_ results in more efficient adsorption, possibly due to the stronger electrostatic interactions, and surface complexation. Similarly, Fe_3_O_4_@SiO_2_-EDTA, which includes the metal-chelating agent EDTA, displayed the lowest adsorption capacities for Pd­(II) and Cu­(II) with 114.94 and 36.9 mg/g, respectively. This result suggests that while EDTA can facilitate metal ion adsorption, the restricted access to chelating sides or lower surface area might limit its overall performance. On the other hand, the magnetic graphene oxide adsorbent showed relatively high adsorption of Cu­(II) (353.59 mg/g) but was still inferior to Ni@Fe_3_O_4_ nano adsorbent. Thus, Ni@Fe_3_O_4_ nano adsorbent results as the most effective nano adsorbent among the reported ones.

## 4 Conclusions and Perspectives

This research concludes that Ni@Fe_3_O_4_ nanoparticles serve as an effective, reusable nanocatalyst and high-performing adsorbent for metal ion removal, particularly for Pd­(II) and Cu­(II). In the synthesis of 1,4-DHP, the Ni@Fe_3_O_4_ catalyst demonstrated excellent catalytic efficiency and reusability, with yields consistently up to 96%. The synthesized 1,4-DHP compounds were also evaluated for their antimicrobial activity, where derivatives containing polar groups at the para position, such as compounds **3d**, **3e**, and **3f**, showed exceptional resistance against tested microbial strains, demonstrating potency comparable to that of standard drugs. For metal ion adsorption, the optimal Ni@Fe_3_O_4_ dosage was determined to be 0.07 g, with a high removal efficiency for Pd­(II) and Cu­(II) observed at a pH of 4, attributed to the protonation effect enhancing the active site interaction. Langmuir and Freundlich isotherm analyses revealed that the nano adsorbent has a significantly higher affinity for Pd­(II) than Cu­(II), with adsorption capacities of 968.18 mg/g for Pd­(II) and 758.99 mg/g for Cu­(II). This work establishes Ni@Fe_3_O_4_ as a dual-purpose material that is not only an efficient catalyst for organic synthesis but also a superior adsorbent for heavy metal remediation, making it promising for both chemical synthesis and environmental applications.

## Supplementary Material



## Notes

Optimization of Ni:Fe molar ratio and its effect on adsorption and catalytic performance (SI-1); characterization of 1,4-DHP derivatives 3a–m (SI-2); and reusability tests (SI-3) (PDF)
