# Supplementary material for: Novel Synergistic Synthesis of Ni-Doped Fe3O4 Nanocomposite: Adsorption Interactions with Heavy Metal Ions and Antimicrobial Studies of Hantzsch Products
Source: ACS Omega. 2025 Sep 16;10(41):48418–32. doi: 10.1021/acsomega.5c05934 (PMC12547568; doi:10.1021/acsomega.5c05934)
Supplement: Supplementary file 1 [file ao5c05934_si_001.pdf]

SUPPORTING INFORMATION SECTION

Novel synergistic synthesis of Ni-doped Fe_3O_4 nanocomposite: Adsorption interactions with heavy metal ions and antimicrobial studies of Hantzsch products

Ashutosh Gupta[1], Priyanshi Kamboj[1], Kaushik Pal[2,*], Jyoti Dagar[1], Aayushi Mandhan[1], Manvinder Kaur[1,3,*], Navneet Kaur[1], Harvinder Singh Sohal[1,*], George Z Kyzas[4,*]

[1] *Department of Chemistry, Chandigarh University, Gharuan-140413, Mohali, Punjab, India*
[2] *University Center for Research and Development (UCRD), Department of Physics, Chandigarh University, Ludhiana - Chandigarh State Hwy, Gharuan-140413, Mohali, Punjab, India*
[3] *Chitkara University Institute of Engineering and Technology, Chitkara University, Rajpura-140401, Punjab, India*
[4] *Hephaestus Laboratory, School of Chemistry, Faculty of Sciences, Democritus University of Thrace, GR 65404 Kavala, Greece*

**Correspondence: kaushikphysics@gmail.com (K.P.); manvinder.2k50@gmail.com (M.K.); drharvinder.cu@gmail.com (H.S.S.); kyzas@chem.duth.gr (G.Z.K.)*

SI1. Optimization of Ni:Fe Molar Ratio and Its Effect on Adsorption and Catalytic Performance

Table S1. Effect of Ni:Fe Molar Ratio on the Adsorption and Catalytic Performance of Ni@Fe_3O_4 Nanoparticles.

Ni:Fe Molar Ratio	Adsorption Capacity of Pd(II) (mg/g)	Adsorption Capacity of Cu(II) (mg/g)	Yield of compound 3a (%)*
1:3	602.22 ±21.22	588.43 ±19.31	72
1:6	968.18 ±29.66	758.99 ±20.74	96
1:9	626.91 ±11.29	614.264 ±10.38	79

*Reaction conditions: Ni@Fe_3O_4 nanoparticles (5 mmol%), and ethanol (10 mL).

As shown in **Table S1**, the Ni@Fe_3O_4 nanoparticles with a Ni:Fe molar ratio of 1:6 exhibited the highest adsorption capacities for Pd(II) (968.18 mg/g) and Cu(II) (758.99 mg/g), along with the best catalytic yield of compound 3a (96%). This performance was attributed to an optimal distribution of Ni active sites and sufficient Fe_3O_4 surface area, enabling effective adsorption and electron transfer. In contrast, a higher Ni ratio (1:3) led to agglomeration and reduced surface availability, while a lower Ni ratio (1:9) limited active sites, resulting in decreased adsorption and catalytic efficiency. Overall, the 1:6 ratio provided the best balance for performance.

SI2. Characterization of 1,4-DHP derivatives 3a-m

3,3,6,6-tetramethyl-9-phenyl-3,4,6,7-tetrahydroacridine-1,8(2H,5H,9H,10H)-dione (3a): Yield 96%, pale yellow crystalline solid, m.p. 280-281ºC [Lit.m.p. 279-280ºC]. IR spectra (ʋ, cm^{-1}): 3402 (N-H), 2956.8 (sp^2, Ar-C-H), 2877, 2831 (sp^3, C-H), 1582.19 (C=O). ^1H NMR spectrum (500 MHz, $CDCl_3$): 11.89 (s, 1H, NH), 7.24-7.27 (d, 2H, Ar-H), 7.10-7.09 (d, 2H, Ar-H), 7.17-7.11 (s, 1H, CH), 2.47-2.22 (m, 8H, CH_2), 1.22-1.67 (s, 6H, CH_3), 1.09-1.06 (s, 6H, CH_3). ^{13}C NMR spectrum (125 MHz, $CDCl_3$): 195.4, 191.6, 188.2, 136.3, 130.9, 127.2, 118.1, 114.2, 77.1, 77.0, 75.3, 46.3, 46.1, 31.4, 30.3, 28.4, 27.6, 27.1.

9-(3-bromophenyl)-3,3,6,6-tetramethyl-3,4,6,7-tetrahydroacridine-1,8(2H,5H,9H,10H)-dione (3b): Yield 95%, pale yellow crystalline solid, m.p. 303-304ºC [Lit.m.p. 305-307ºC]. IR spectra (ʋ, cm^{-1}): 3089 (N-H), 2962.2 (sp^2, Ar-C-H), 2867.4 (sp^3, C-H), 1579 (C=O). ^1H NMR spectrum (500 MHz, $CDCl_3$): 11.79 (s, 1H, NH), 7.97-7.72 (d, 4H, Ar-H), 5.53 (s, 1H, CH),

2.50-2.31 (m, 8H, CH_2), 1.23 (s, 6H, CH_3), 1.11 (s, 6H, CH_3). ^{13}C NMR spectrum (125 MHz, $CDCl_3$): 189.9, 189.5, 185.2, 137.1, 130.5, 128.2, 119.3, 115.1, 77.2, 76.9, 76.2, 45.9. 31.5, 31.1, 29.2, 27.9, 27.2.

9-(4-bromophenyl)-3,3,6,6-tetramethyl-3,4,6,7-tetrahydroacridine-1,8(2H,5H,9H,10H)-dione (3c): Yield 96%, pale yellow crystalline solid, m.p. 239-241ºC [Lit.m.p. 238-240ºC]. IR spectra (ʋ, cm^{-1}): 3210 (N-H), 2966.5 (sp^2, Ar-C-H), 2879.2 (sp^3, C-H), 1619.38 (C=O). ^1H NMR spectrum (500 MHz, $CDCl_3$): 11.86 (s, 1H, NH), 7.37-7.34 (d, 2H, Ar-H), 6.96-6.94 (d, 2H, Ar-H), 5.44 (s, 1H, CH), 2.46-2.22 (m, 8H, CH_2), 1.21 (s, 6H, CH_3), 1.09 (s, 6H, CH_3). ^{13}C NMR spectrum (125 MHz, $CDCl_3$): 197.5, 190.6, 189.4, 137.3, 131.2, 128.6, 119.5, 115.2, 77.3, 77.1, 76.8, 46.9, 46.4, 32.4, 31.4, 29.5, 28.0, 27.4.

3,3,6,6-tetramethyl-9-(3-nitrophenyl)-3,4,6,7-tetrahydroacridine-1,8(2H,5H,9H,10H)-dione (3d): Yield 96%, pale yellow crystalline solid, m.p. 275-276ºC [Lit.m.p. 273-275ºC]. IR spectra (ʋ, cm^{-1}): 3168.5 (N-H), 2952.8 (sp^2, Ar-C-H), 2886 (sp^3, C-H), 1590.90 (C=O). ^1H NMR spectrum (500 MHz, $CDCl_3$): 11.85 (s, 1H, NH), 8.00-8.05 (d, 2H, Ar-H), 7.45-7.27 (d, 2H, Ar-H), 5.54 (s, 1H, CH), 2.51-2.32 (m, 8H, CH_2), 1.27 (s, 6H, CH_3), 1.12 (s, 6H, CH_3). ^{13}C NMR spectrum (125 MHz, $CDCl_3$): 191.1, 189.6, 148.4, 140.7, 132.9, 129.1, 122.2, 121.0, 114.7, 77.3, 77.1, 76.8, 46.9, 46.4, 32.9, 31.4, 29.6, 27.7.

3,3,6,6-tetramethyl-9-(4-nitrophenyl)-3,4,6,7-tetrahydroacridine-1,8(2H,5H,9H,10H)-dione (3e): Yield 95%, pale yellow crystalline solid, m.p. 280-281ºC [Lit.m.p. 282-283ºC]. IR spectra (ʋ, cm^{-1}): 3089.3 (N-H), 2961.3 (sp^2, Ar-C-H), 2869.5 (sp^3, C-H), 1592.81 (C=O). ^1H NMR spectrum (500 MHz, $CDCl_3$): 11.79 (s, 1H, NH), 8.14-8.11 (d, 2H, Ar-H), 7.28-7.23 (s, 2H, Ar-H), 5.54 (s, 1H, CH), 2.51-2.32 (m, 8H, CH_2), 1.23 (s, 6H, CH_3), 1.11 (s, 6H, CH_3). ^{13}C NMR spectrum (125 MHz, $CDCl_3$): 190.1, 189.5, 187.3, 146.5, 146.1, 127.6, 123.5, 114.9, 77.3, 77.0, 76.8, 46.9, 46.4, 33.2, 31.5, 29.5, 27.4, 27.1.

9-(2-chlorophenyl)-3,3,6,6-tetramethyl-3,4,6,7-tetrahydroacridine-1,8(2H,5H,9H,10H)-dione (3f): Yield 93%, pale yellow crystalline solid, m.p. 219-220ºC [Lit.m.p. 217-219ºC]. IR spectra (ʋ, cm^{-1}): 3434.2 (N-H), 2964.3 (sp^2, Ar-C-H), 2898 (sp^3, C-H), 1587 (C=O). ^1H NMR spectrum (500 MHz, $CDCl_3$): 11.70 (s, 1H, NH), 7.17-7.13 (d, 1H, Ar-H), 7.02-6.99 (s, 3H, Ar-H), 5.46 (s, 1H, CH), 2.69-2.34 (m, 8H, CH_2), 1.26 (s, 6H, CH_3), 0.99 (s, 6H, CH_3). ^{13}C NMR

spectrum (125 MHz, $CDCl_3$): 190.4, 190.2, 189.4, 146.3, 143.6, 129.7, 119.5, 115.8, 114.1, 109.8, 55.7, 47.1, 46.4, 32.3, 31.2, 29.8, 27.1, 27.0.

9-(4-chlorophenyl)-3,3,6,6-tetramethyl-3,4,6,7-tetrahydroacridine-1,8(2H,5H,9H,10H)-dione (3g): Yield 94%, pale yellow crystalline solid, m.p. 227-228ºC [Lit.m.p. 228-229ºC]. IR spectra (ʋ, cm^{-1}): 3216.77 (N-H), 2982.1 (Ar-C-H), 2919, 2887 (sp^3, C-H), 1505.16 (C=O). ^1H NMR spectrum (500 MHz, $CDCl_3$): 11.88 (s, 1H, NH), 7.85-7.79 (d, 4H, Ar-H), 5.26 (s, 1H, Ar-H), 2.60-2.41 (s, 7H, Ar-H), 1.23 (s, 6H, CH_3), 1.11 (s, 6H, CH_3). ^{13}C NMR spectrum (125 MHz, $CDCl_3$): 192.4, 192.2, 151.5, 142.1, 135.6, 127.9, 126.1, 124.2, 77.2, 77.1, 75.4, 49.4, 46.2, 38.5, 30.1, 29.1, 26.3, 26.0.

9-(2-hydroxyphenyl)-3,3,6,6-tetramethyl-3,4,6,7,9,10-hexahydroacridine-1,8(2H,5H)-dione (3h): Yield 95%, pale yellow crystalline solid, m.p. 307-308ºC [Lit.m.p. 305-306ºC]. IR spectra (ʋ, cm^{-1}): 3322.2 (N-H), 2953.3 (sp^2, Ar-C-H), 2895 (sp^3, C-H), 1567 (C=O). ^1H NMR spectrum (500 MHz, $CDCl_3$): 10.47 (s, 1H, NH), 7.17-7.13 (d, 1H, Ar-H), 7.02-6.99 (m, 3H, Ar-H), 4.67 (s, 1H, CH), 2.61-1.97 (m, 8H, CH_2), 1.12 (s, 3H, CH_3), 1.02 (s, 3H, CH_3), 0.99 (s, 6H, CH_3). ^{13}C NMR spectrum (125 MHz, $CDCl_3$): 200.1, 169.1, 151.1, 127.9, 127.5, 124.5, 124.3, 118.3, 115.7, 111.1, 77.3, 77.1, 76.8, 49.9, 41.6, 32.3, 30.9, 29.2, 27.7, 27.2.

9-(4-methoxyphenyl)-3,3,6,6-tetramethyl-3,4,6,7,9,10-hexahydroacridine-1,8(2H,5H)-dione (3i): Yield 96%, pale yellow crystalline solid, m.p. 305-306ºC [Lit.m.p. 303-305ºC]. IR spectra (ʋ, cm^{-1}): 3327.77 (N-H), 3100.1 (sp^2, Ar-C-H), 2945, 2920 (sp^3 C-H), 1591.16 (C=O). ^1H NMR spectrum (500 MHz, $CDCl_3$): 11.91 (s, 1H, NH), 7.92-7.89 (d, 2H, Ar-H), 7.03-7.00 (d, 2H, Ar-H), 5.39 (s, 1H, CH), 3.92 (s, 3H, OCH_3), 2.41-2.22 (m, 8H, CH_2), 1.20 (s, 6H, CH_3), 1.15 (s, 6H, CH_3). ^{13}C NMR spectrum (125 MHz, $CDCl_3$): 195.3, 189.2, 186.3, 153.4, 147.4, 126.2, 126.1, 125.8, 75.3, 74.9, 70.2, 48.4, 48.0, 39.3, 33.6, 29.8, 26.7, 26.3.

3,3,6,6-tetramethyl-9-p-tolyl-3,4,6,7-tetrahydroacridine-1,8(2H,5H,9H,10H)-dione (3j): Yield 95%, pale yellow crystalline solid, m.p. 240-242ºC. IR spectra (ʋ, cm^{-1}): 3116.77 (N-H), 2953.1 (sp^2, Ar-C-H), 2945, 2927 (sp^3 C-H), 1601.16 (C=O). ^1H NMR spectrum (500 MHz, $CDCl_3$): 11.89 (s, 1H, NH), 7.81-7.72 (d, 2H, Ar-H), 7.34-7.26 (d, 2H, Ar-H), 5.29 (s, 1H, CH), 2.50-2.32 (d, 8H, CH_2), 2.22 (s, 3H, CH_3), 1.23 (s, 6H, CH_3), 1.11 (s, 6H, CH_3). ^{13}C NMR

spectrum (125 MHz, $CDCl_3$): 190.4, 190.3, 154.5, 146.3, 127.5, 127.2, 125.4, 125.1, 71.6, 71.2, 68.5, 47.8, 46.5, 31.4, 29.4, 28.9, 20.9, 20.3.

9-(4-hydroxy-3-methoxyphenyl)-3,3,6,6-tetramethyl-3,4,6,7,9,10-hexahydroacridine-1,8(2H,5 H)-dione (3k): Yield 91%, pale yellow crystalline solid, m.p. 232-234ºC. IR spectra (ʋ, cm^{-1}): 3232.2 (N-H), 2953.1 (sp^2, Ar-C-H), 2794 (sp^3, C-H), 1578 (C=O). ^1H NMR spectrum (500 MHz, $CDCl_3$): 11.96 (s, 1H, NH), 6.80-6.79 (d, 1H, Ar-H), 6.61-6.58 (s, 1H, Ar-H), 6.57-6.56 (d, 1H, Ar-H), 5.49 (s, 1H, CH), 3.76 (s, 3H, OCH_3), 2.43-2.29 (m, 8H, CH_2), 1.23 (s, 6H, CH_3), 1.10 (s, 6H, CH_3). ^{13}C NMR spectrum (125 MHz, $CDCl_3$): 190.4, 189.3, 146.3, 143.6, 129.7, 119.5, 115.8, 114.1, 109.8, 77.3, 77.1, 76.8, 55.7, 47.1, 46.4, 32.3, 31.2, 29.8, 27.1.

9-(furan-2-yl)-3,3,6,6-tetramethyl-3,4,6,7-tetrahydroacridine-1,8(2H,5H,9H,10H)-dione (3l): Yield 91%, pale yellow crystalline solid, m.p. 230ºC. IR spectra (ʋ, cm^{-1}): 3310.77 (N-H), 3025.1 (sp^2, Ar-C-H), 2969, 2930 (sp^3 C-H), 1610.26 (C=O). ^1H NMR spectrum (500 MHz, $CDCl_3$): 11.93 (s, 1H, NH), 7.82-7.81 (d, 1H, Ar-H), 7.54 (m, 1H, Ar-H), 7.37-7.36 (d, 1H, Ar-H), 5.03 (s, 1H, CH), 2.52-2.32 (m, 8H, CH_2), 1.12 (s, 6H, CH_3), 1.02 (s, 6H, CH_3). ^{13}C NMR spectrum (125 MHz, $CDCl_3$): 194.4, 190.1, 154.5, 146.4, 149.3, 123.4, 121.6, 121.3, 75.3, 75.1, 73.5, 49.5, 49.4, 38.5, 31.3, 29.4, 26.6, 26.1.

3,3,6,6-tetramethyl-9-(thiophen-2-yl)-3,4,6,7-tetrahydroacridine-1,8(2H,5H,9H,10H)-dione (3m): Yield 93%, pale yellow crystalline solid, m.p. 237-238ºC [Lit.m.p. 236-238ºC]. IR spectra (ʋ, cm^{-1}): 3226.77 (N-H), 2956.1 (sp^2, Ar-C-H), 2859, 2829 (sp^3 C-H), 1615.16 (C=O). ^1H NMR spectrum (500 MHz, $CDCl_3$): 12.32 (s, 1H, NH), 7.1-7.09 (dd, 1H, Ar-H), 6.87-6.85 (dd, 1H, Ar-H), 6.64-6.63 (d, 1H, Ar-H), 5.63 (s, 1H, CH), 2.43-2.23 (m, 8H, CH_2), 1.21 (s, 6H, CH_3), 1.09 (s, 6H, CH_3). ^{13}C NMR spectrum (125 MHz, $CDCl_3$): 189.9, 189.5, 143.7, 1236.3, 124.5, 123.4, 115.9, 77.3, 77.1, 76.8, 46.9, 46.2, 31.1, 30.4, 29.9, 26.7.

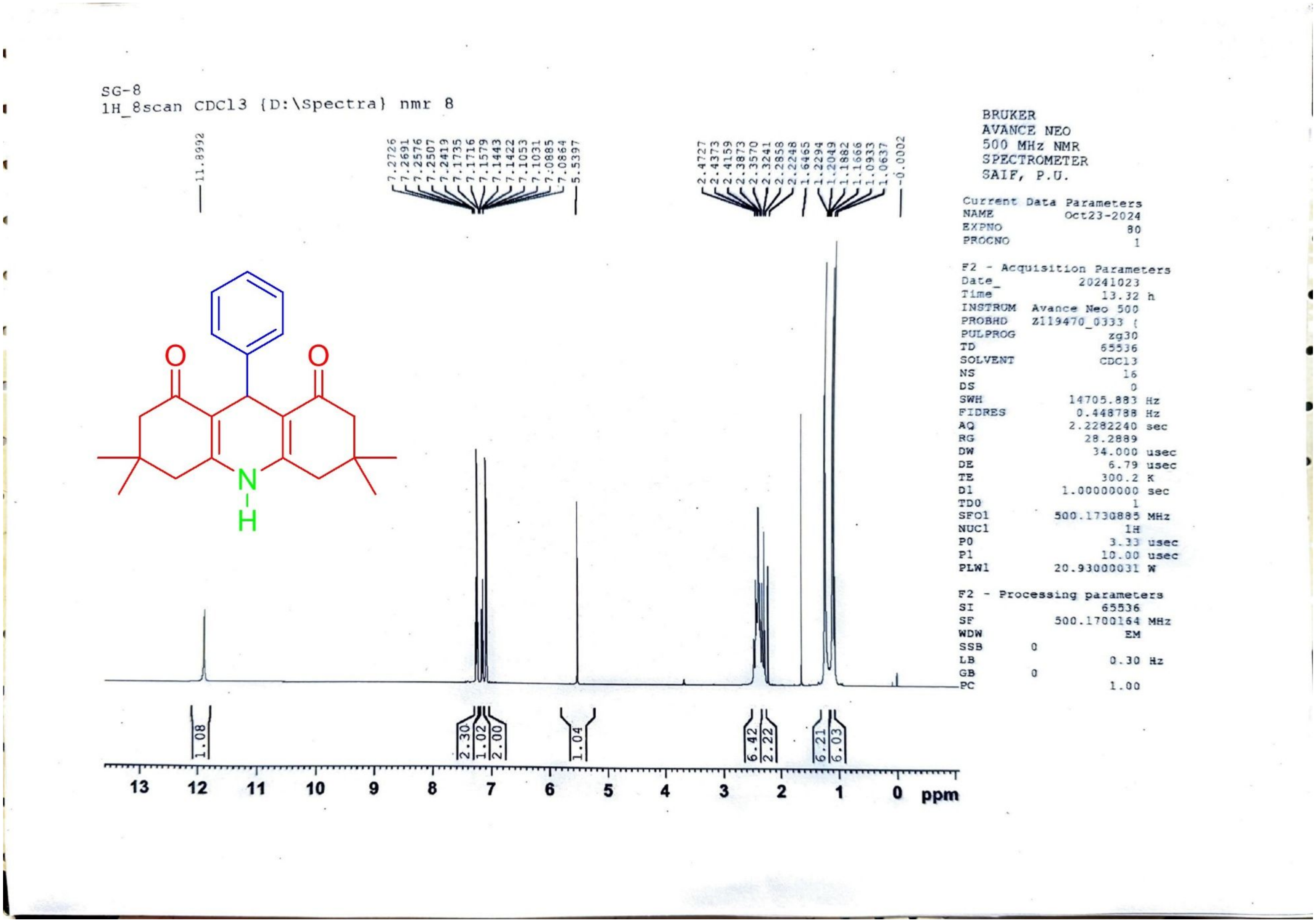

Figure S1. ^{1}H NMR spectrum of 3,3,6,6-tetramethyl-9-phenyl-3,4,6,7-tetrahydroacridine-1,8(2H,5H,9H,10H)-dione (3a).

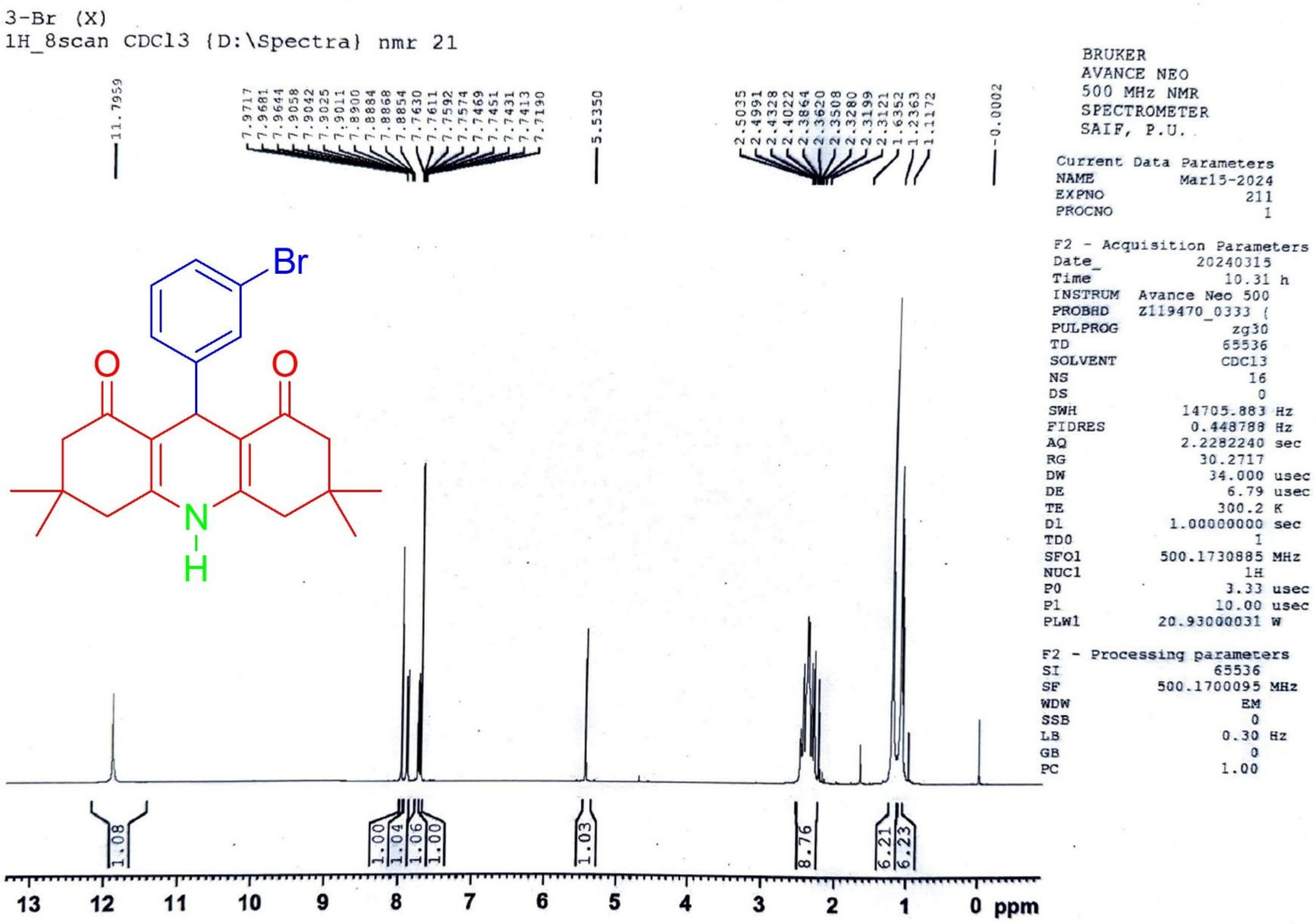

Figure S2. ^{1}H NMR spectrum of 9-(3-bromophenyl)-3,3,6,6-tetramethyl-3,4,6,7-tetrahydroacridine-1,8(2H,5H,9H,10H)-dione (3b).

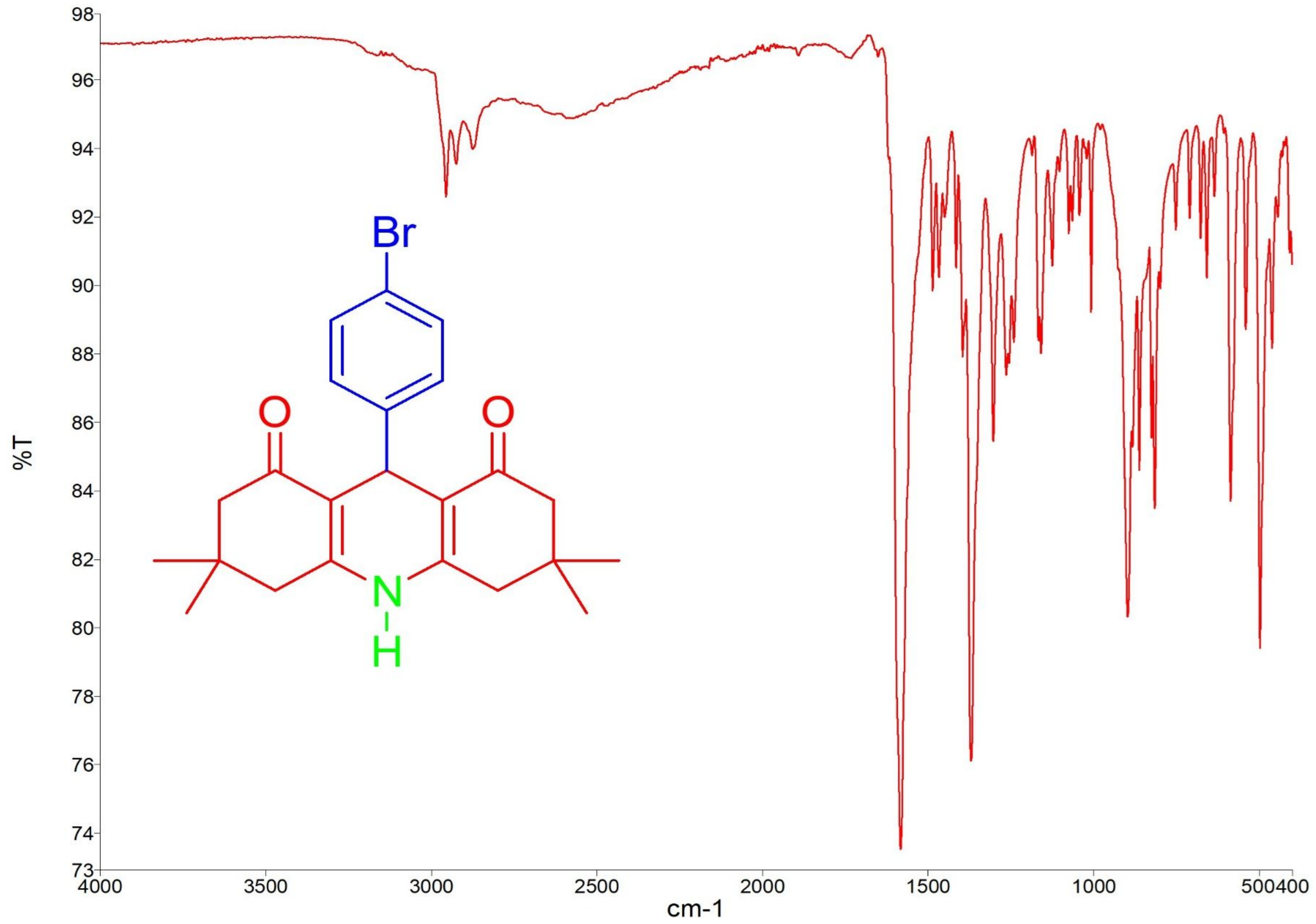

Figure S3. IR spectrum of 9-(4-bromophenyl)-3,3,6,6-tetramethyl-3,4,6,7-tetrahydroacridine-1,8(2H,5H,9H,10H)-dione (3c).

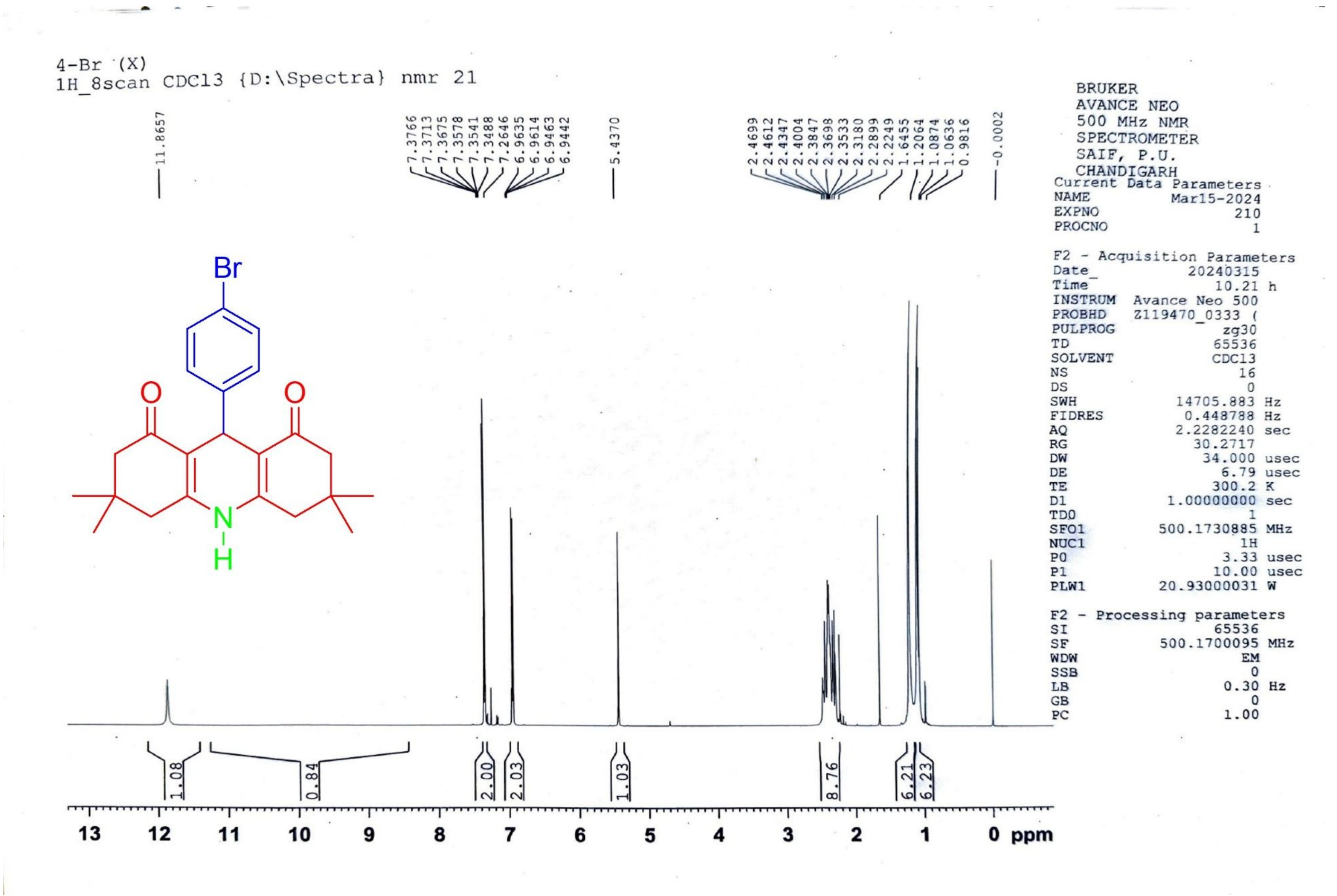

Figure S4. ^{1}H NMR spectrum of 9-(4-bromophenyl)-3,3,6,6-tetramethyl-3,4,6,7-tetrahydroacridine-1,8(2H,5H,9H,10H)-dione (3c).

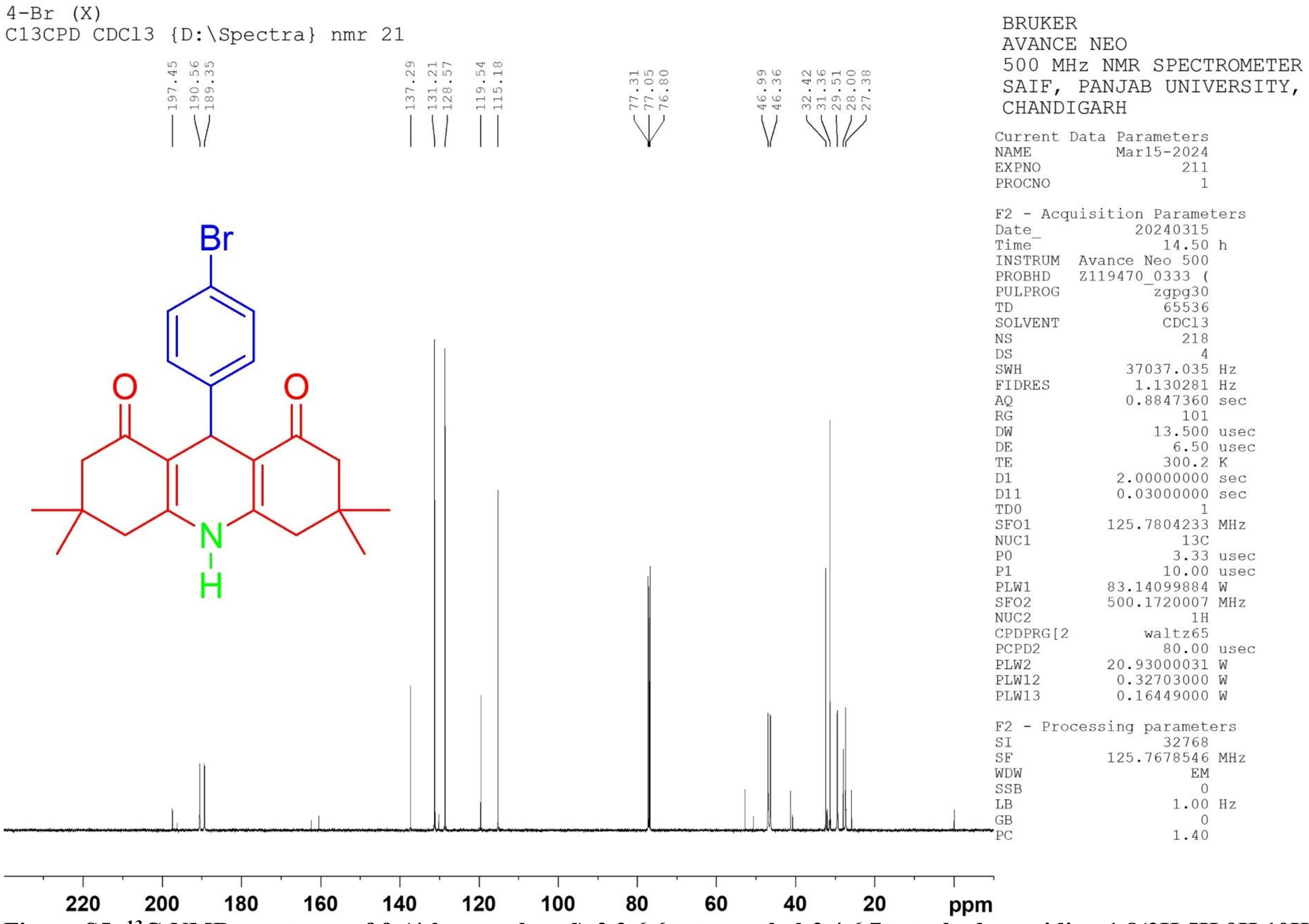

Figure S5. ^{13}**C NMR spectrum of 9-(4-bromophenyl)-3,3,6,6-tetramethyl-3,4,6,7-tetrahydroacridine-1,8(2H,5H,9H,10H)-dione (3c).**

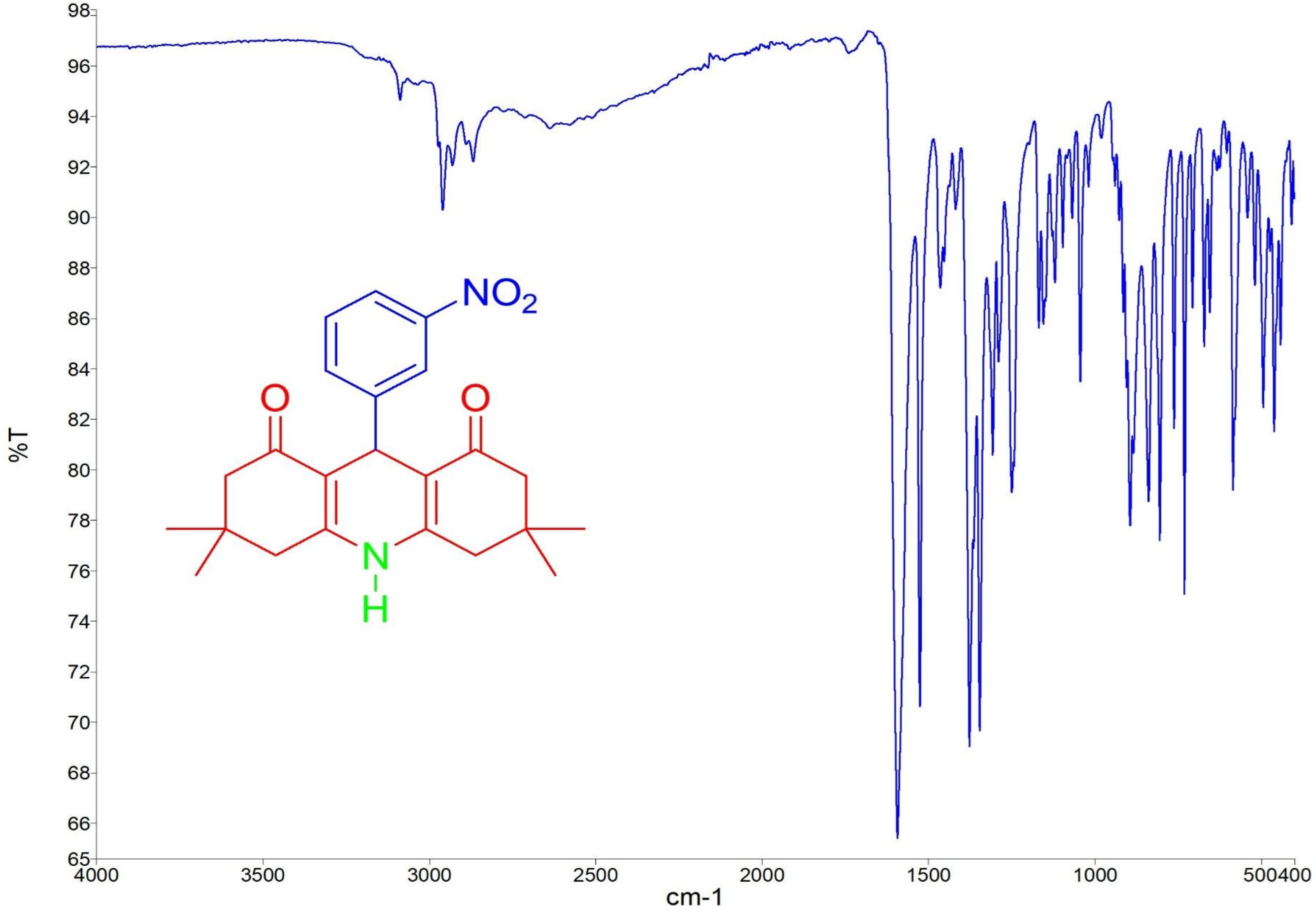

Figure S6. IR spectrum of 3,3,6,6-tetramethyl-9-(3-nitrophenyl)-3,4,6,7-tetrahydroacridine-1,8(2H,5H,9H,10H)-dione (3d).

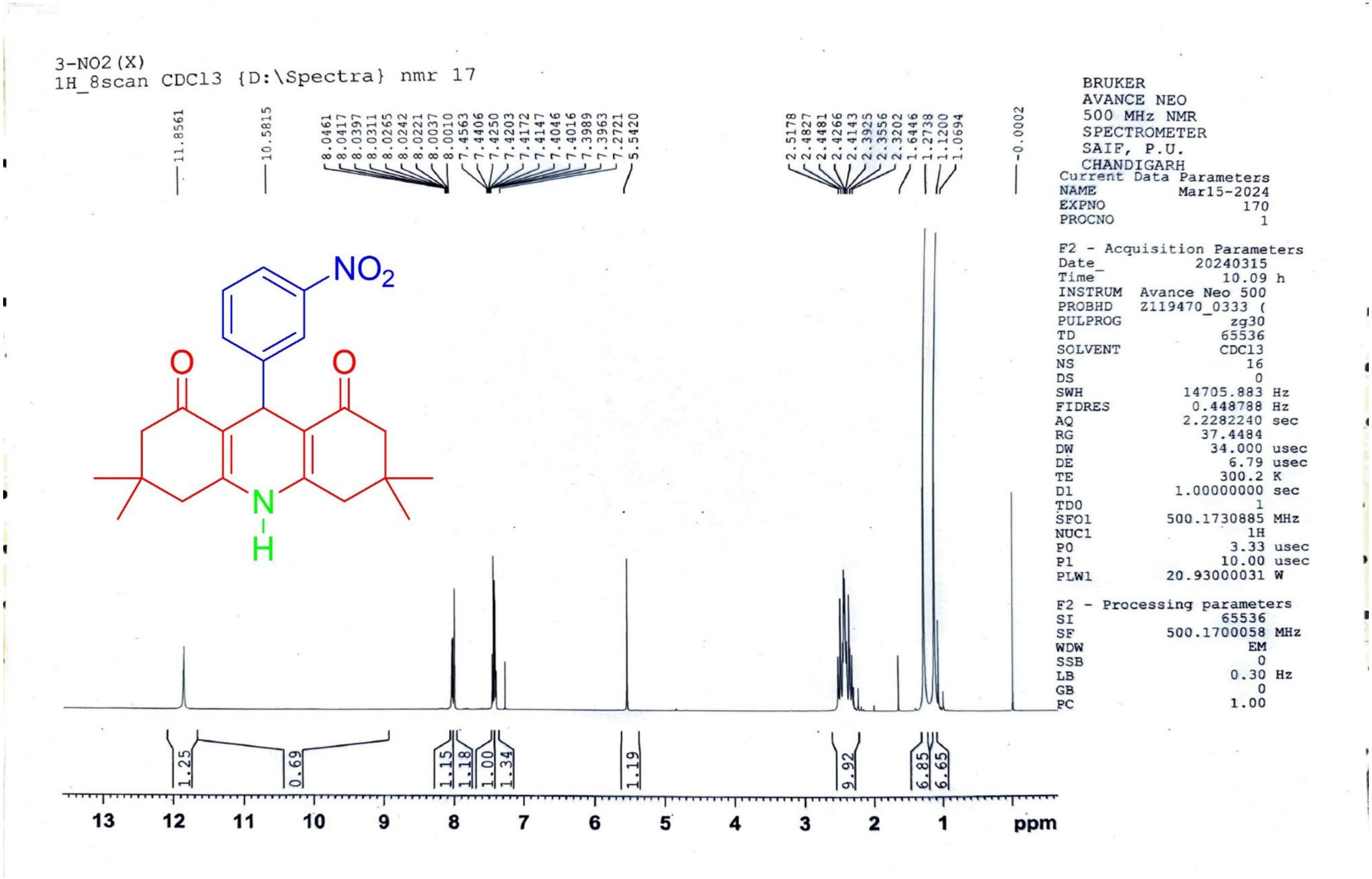

Figure S7. ^{1}**H NMR spectrum of 3,3,6,6-tetramethyl-9-(3-nitrophenyl)-3,4,6,7-tetrahydroacridine-1,8(2H,5H,9H,10H)-dione (3d).**

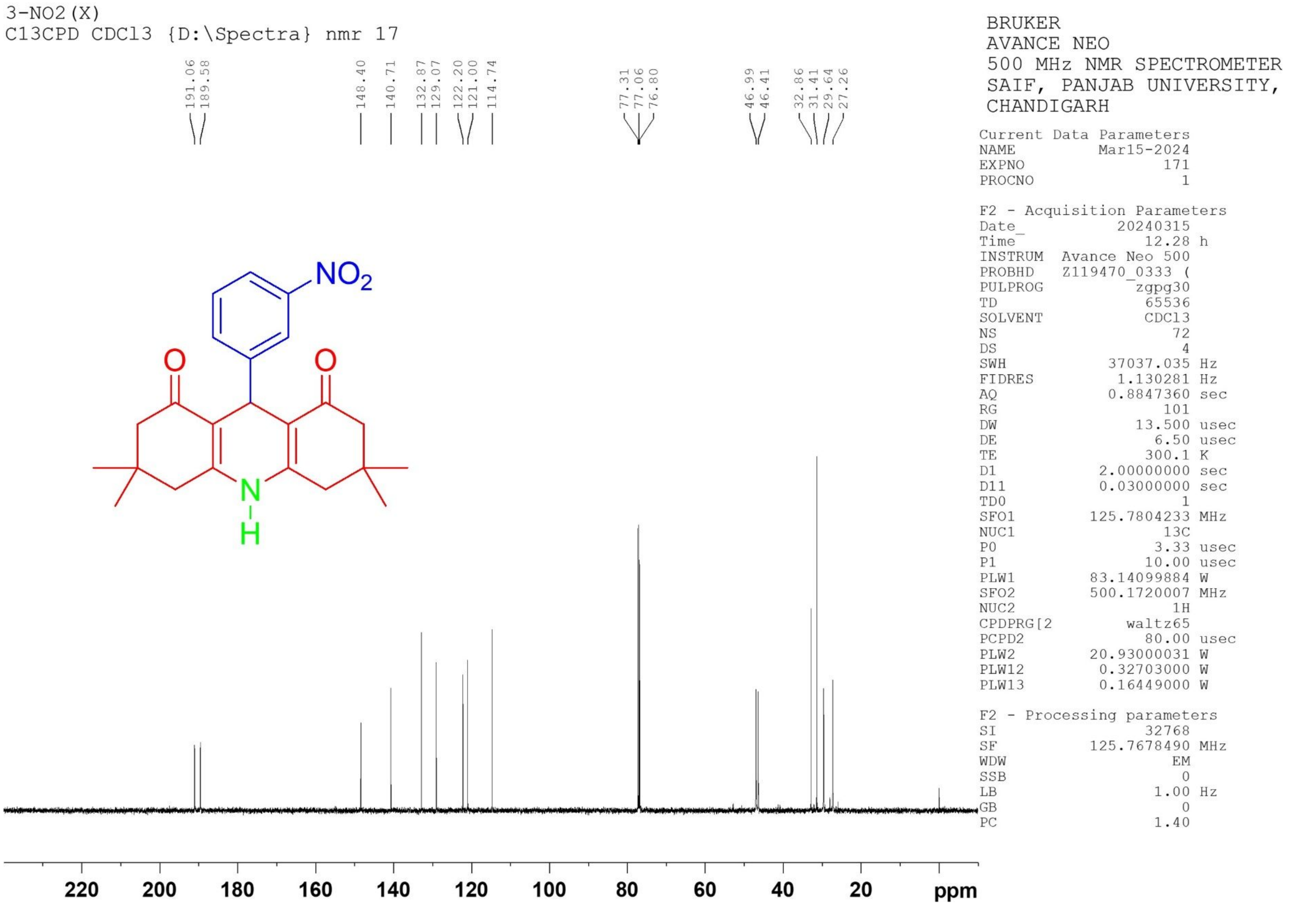

Figure S8. ^{13}C NMR spectrum of 3,3,6,6-tetramethyl-9-(3-nitrophenyl)-3,4,6,7-tetrahydroacridine-1,8(2H,5H,9H,10H)-dione (3d).

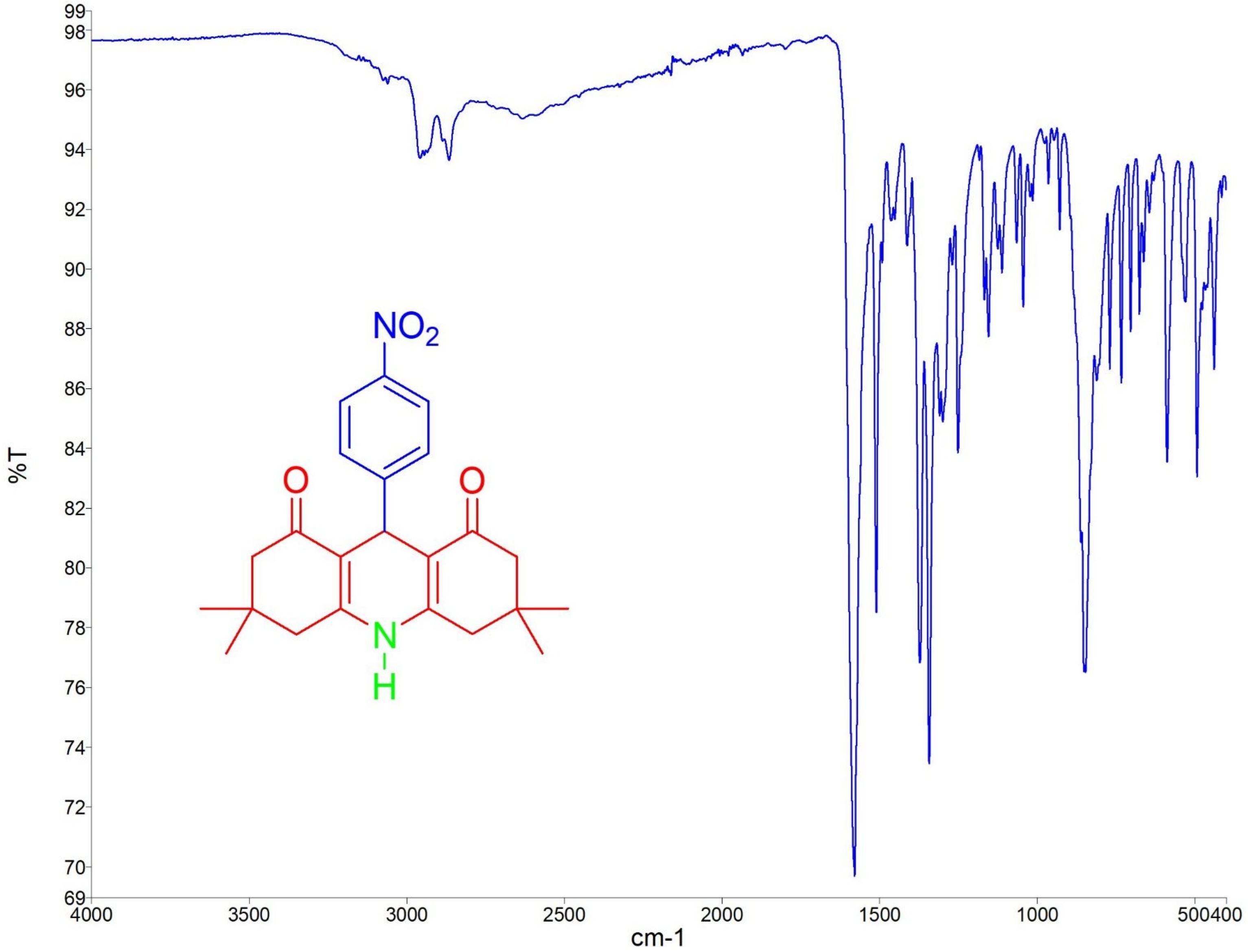

Figure S9. IR spectrum of 3,3,6,6-tetramethyl-9-(4-nitrophenyl)-3,4,6,7-tetrahydroacridine-1,8(2H,5H,9H,10H)-dione (3e).

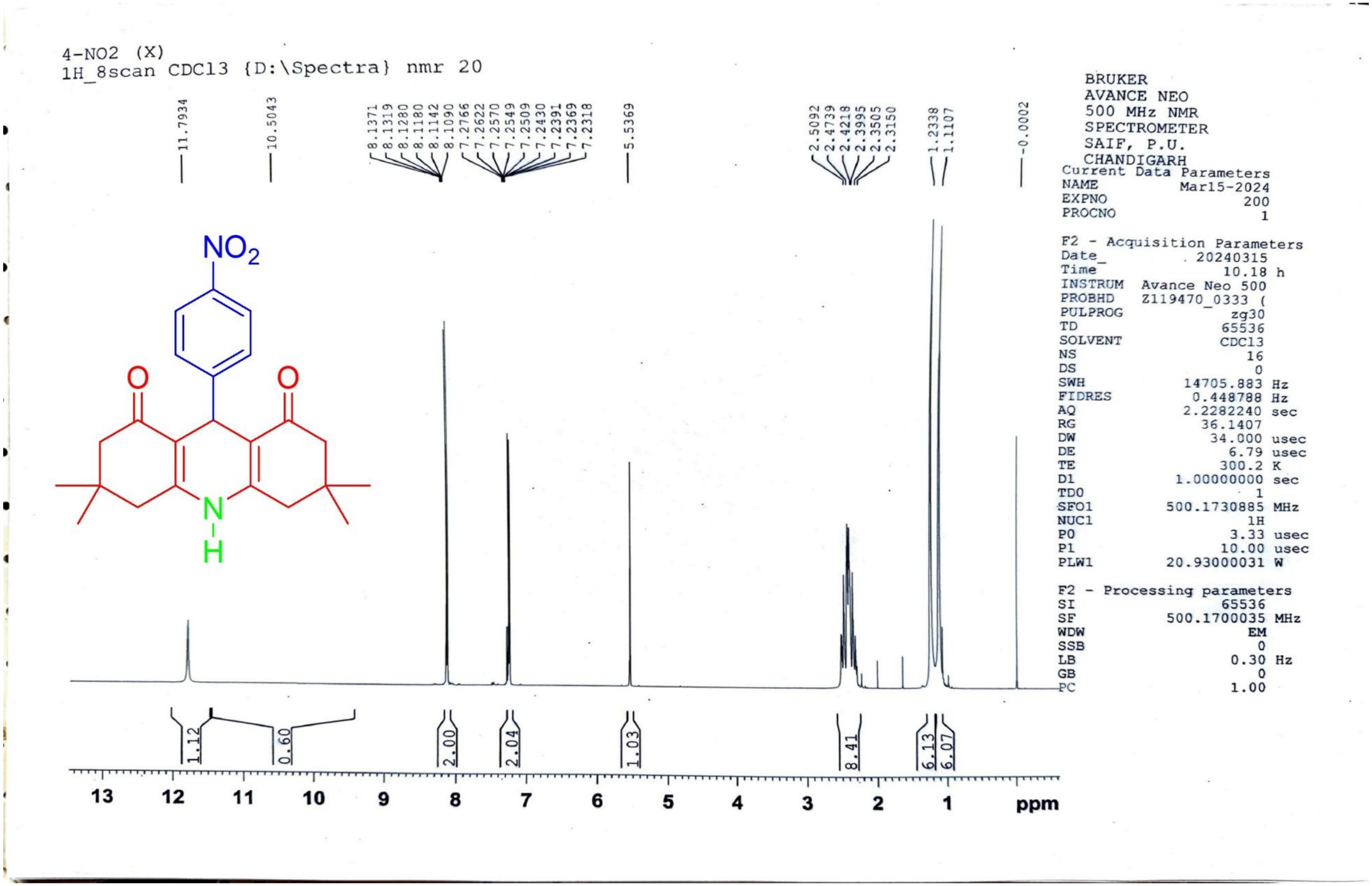

Figure S10. **^{1}H NMR spectrum of 3,3,6,6-tetramethyl-9-(4-nitrophenyl)-3,4,6,7-tetrahydroacridine-1,8(2H,5H,9H,10H)-dione (3e).**

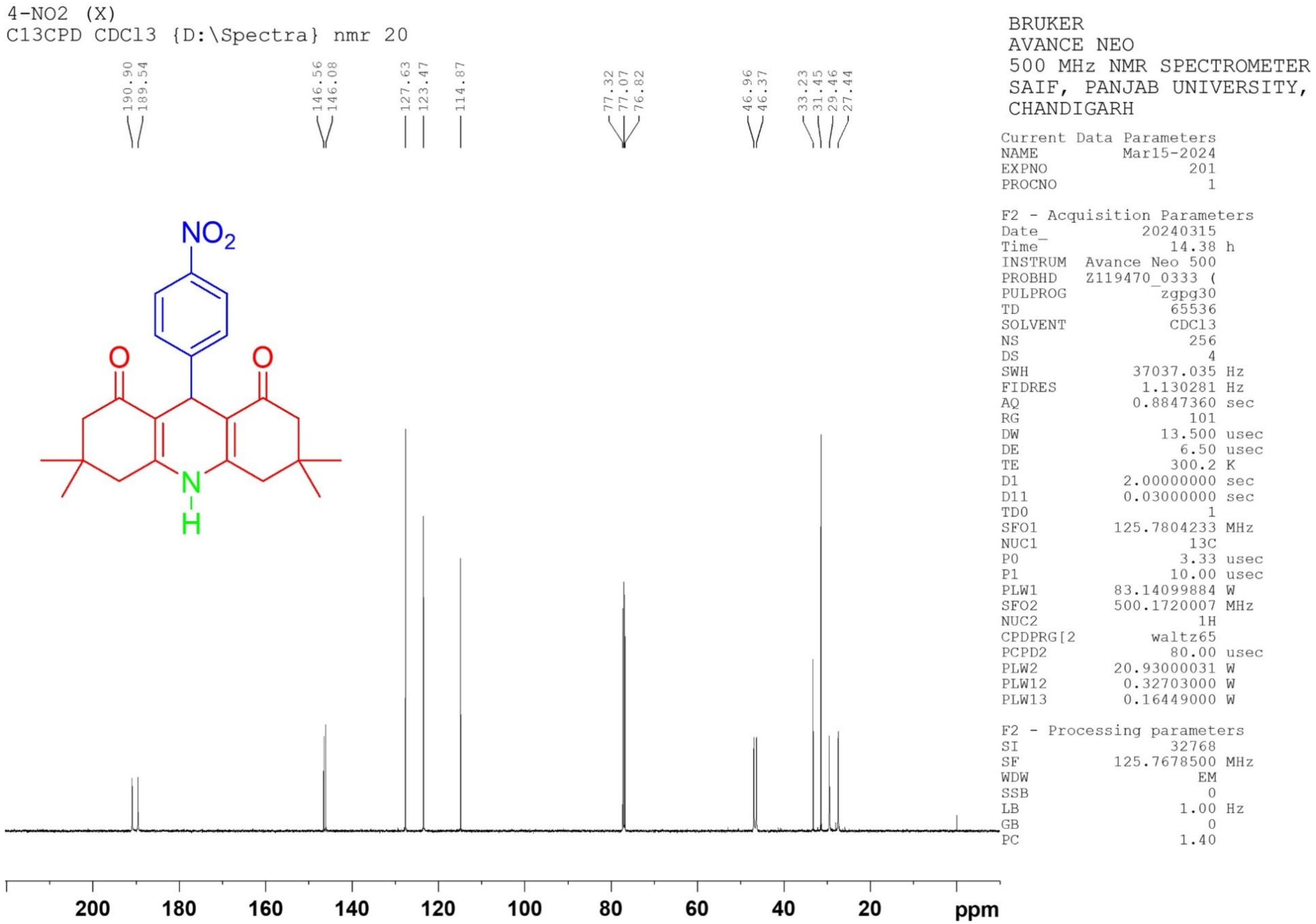

Figure S11. ^{13}C **NMR spectrum of 3,3,6,6-tetramethyl-9-(4-nitrophenyl)-3,4,6,7-tetrahydroacridine-1,8(2H,5H,9H,10H)-dione (3e).**

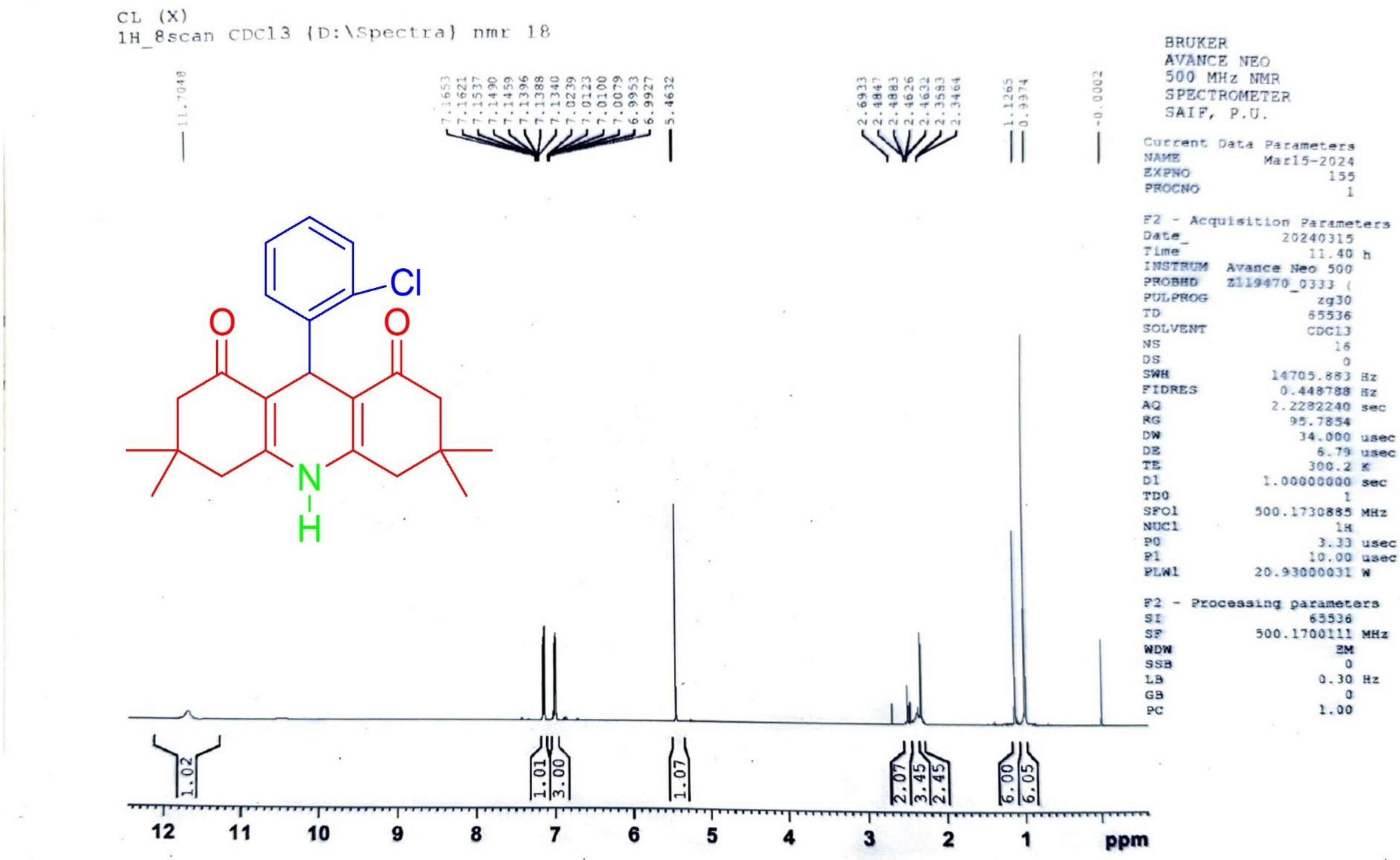

Figure S12. **^{1}H NMR spectrum of 9-(2-chlorophenyl)-3,3,6,6-tetramethyl-3,4,6,7-tetrahydroacridine-1,8(2H,5H,9H,10H)-dione (3f).**

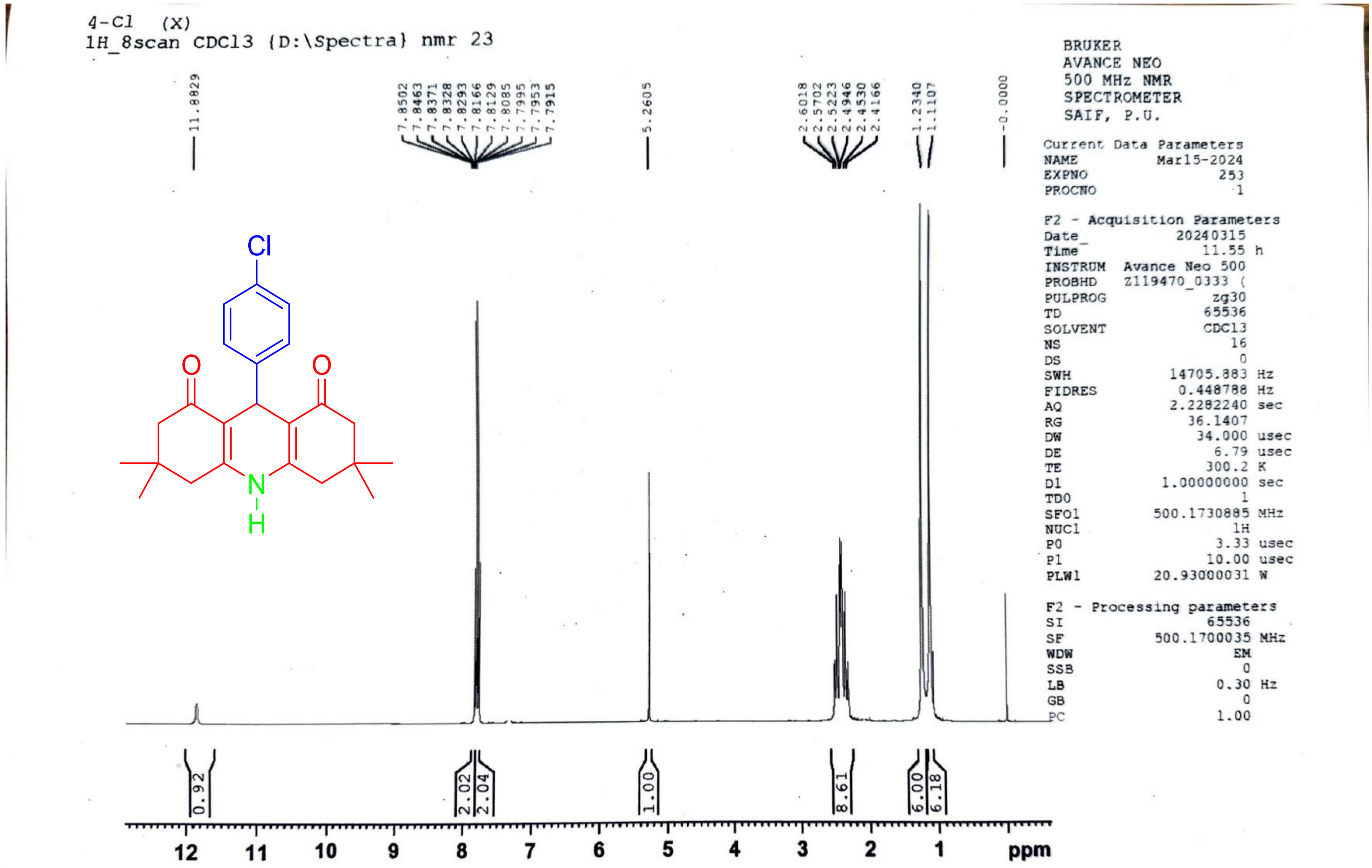

Figure S13. ^{1}H **NMR spectrum of 9-(4-chlorophenyl)-3,3,6,6-tetramethyl-3,4,6,7-tetrahydroacridine-1,8(2H,5H,9H,10H)-dione (3g).**

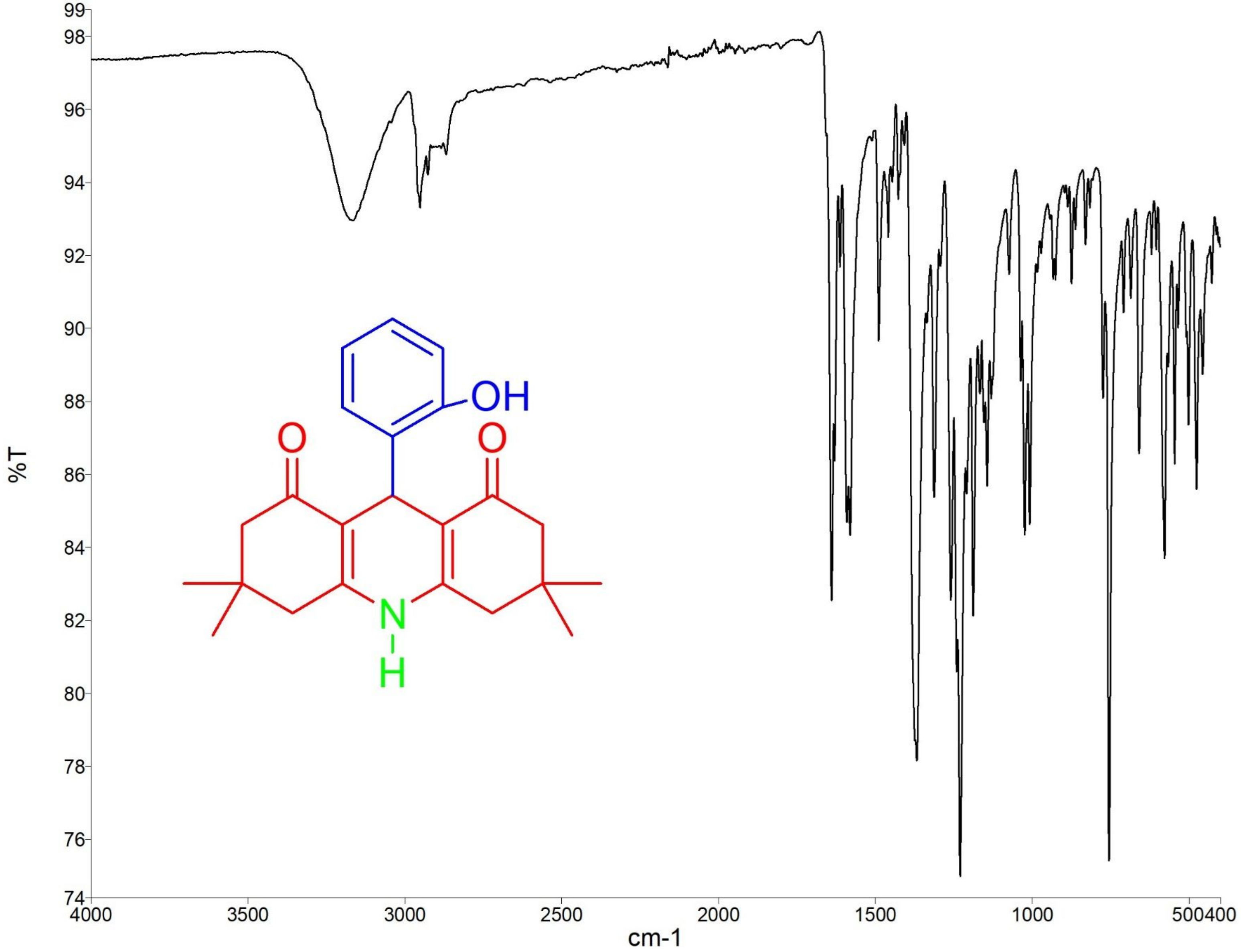

Figure S14. IR spectrum of 9-(2-hydroxyphenyl)-3,3,6,6-tetramethyl-3,4,6,7,9,10-hexahydroacridine-1,8(2H,5H)-dione (3h).

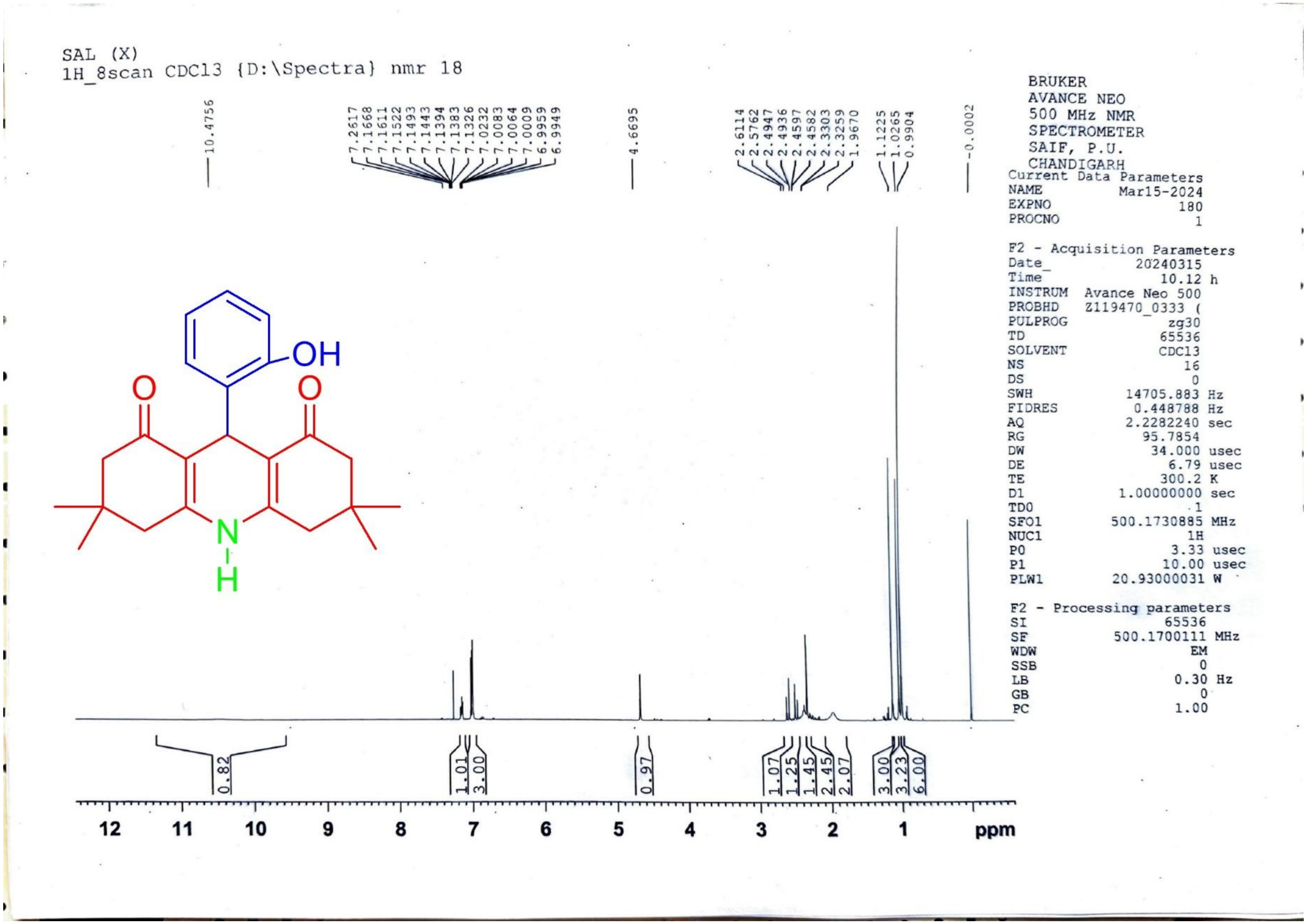

Figure S15. ^{1}H **NMR spectrum of 9-(2-hydroxyphenyl)-3,3,6,6-tetramethyl-3,4,6,7,9,10-hexahydroacridine-1,8(2H,5H)-dione (3h).**

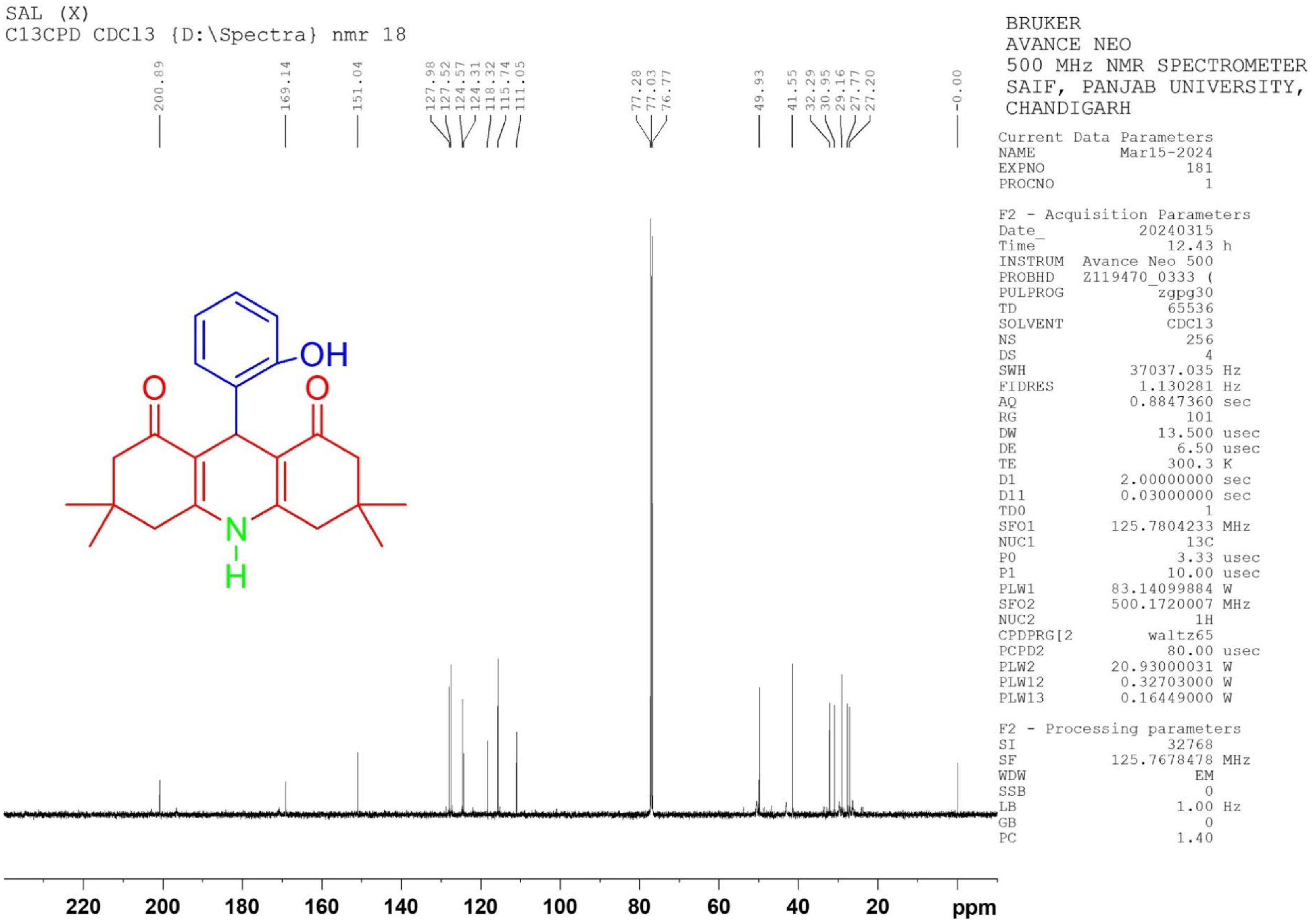

Figure S16. ^{13}**C NMR spectrum of 9-(2-hydroxyphenyl)-3,3,6,6-tetramethyl-3,4,6,7,9,10-hexahydroacridine-1,8(2H,5H)-dione (3h).**

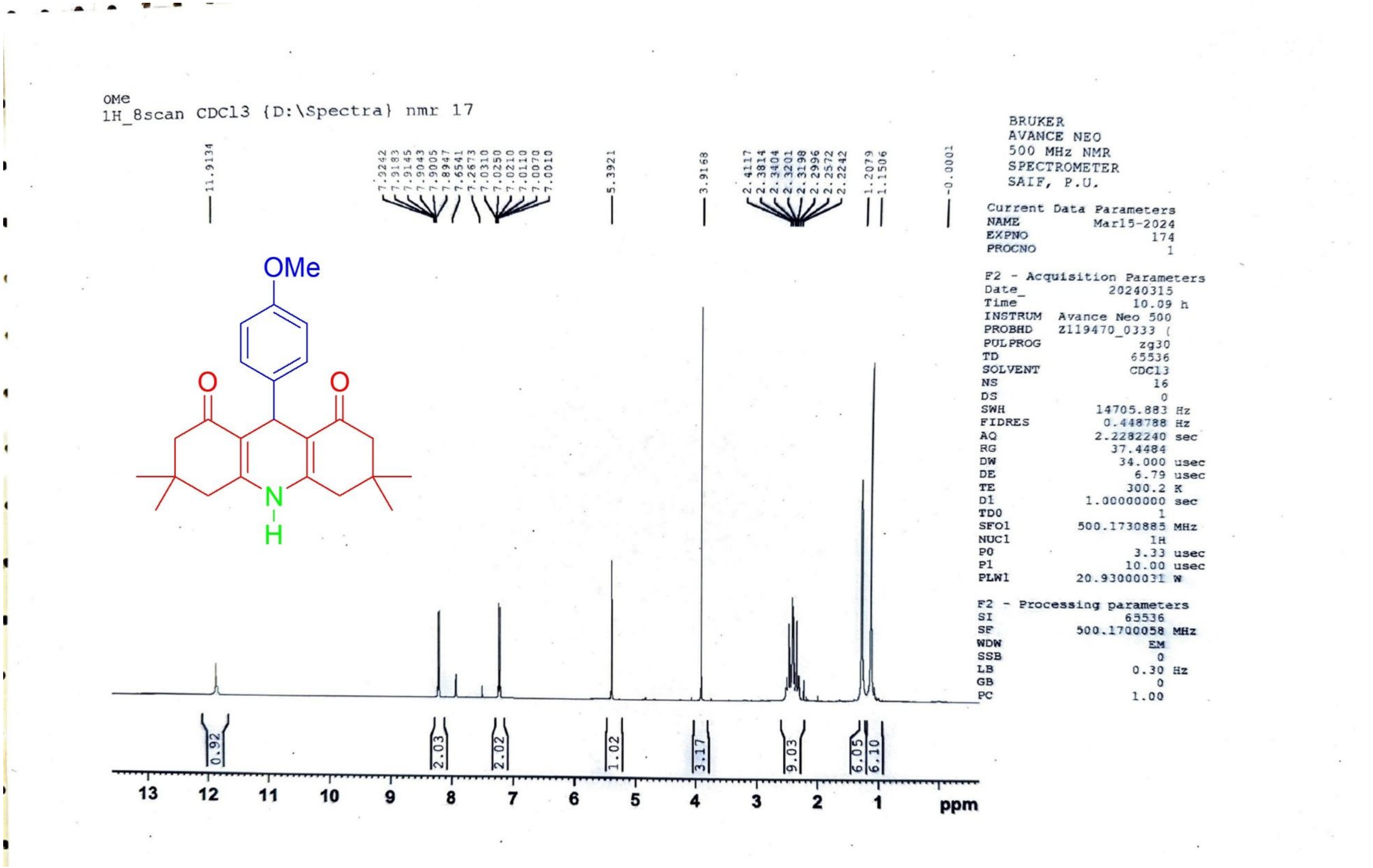

Figure S17. **^{1}H NMR spectrum of 9-(4-methoxyphenyl)-3,3,6,6-tetramethyl-3,4,6,7,9,10-hexahydroacridine-1,8(2H,5H)-dione (3i).**

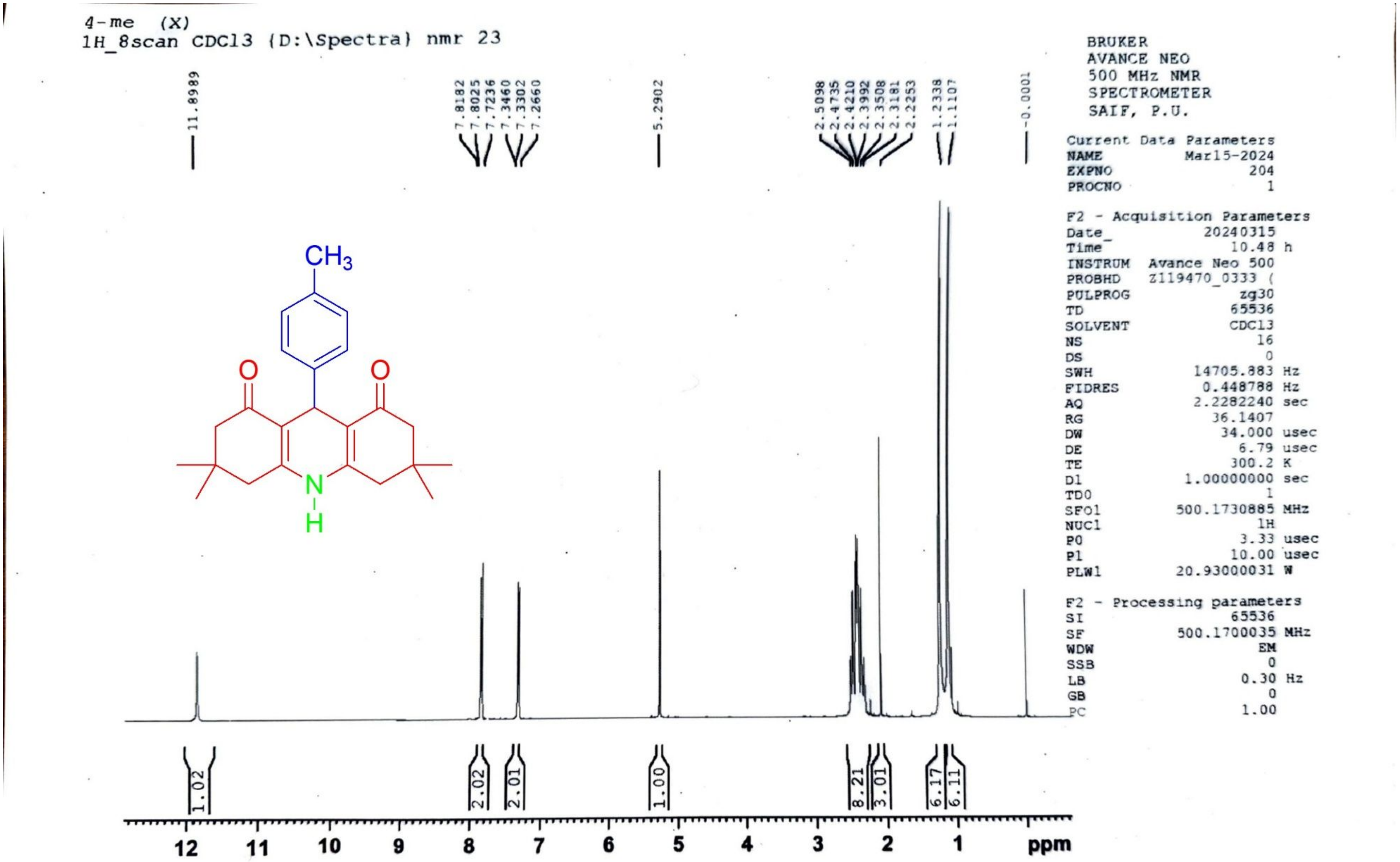

Figure S18. ¹H NMR spectrum of 3,3,6,6-tetramethyl-9-p-tolyl-3,4,6,7-tetrahydroacridine-1,8(2H,5H,9H,10H)-dione (3j).

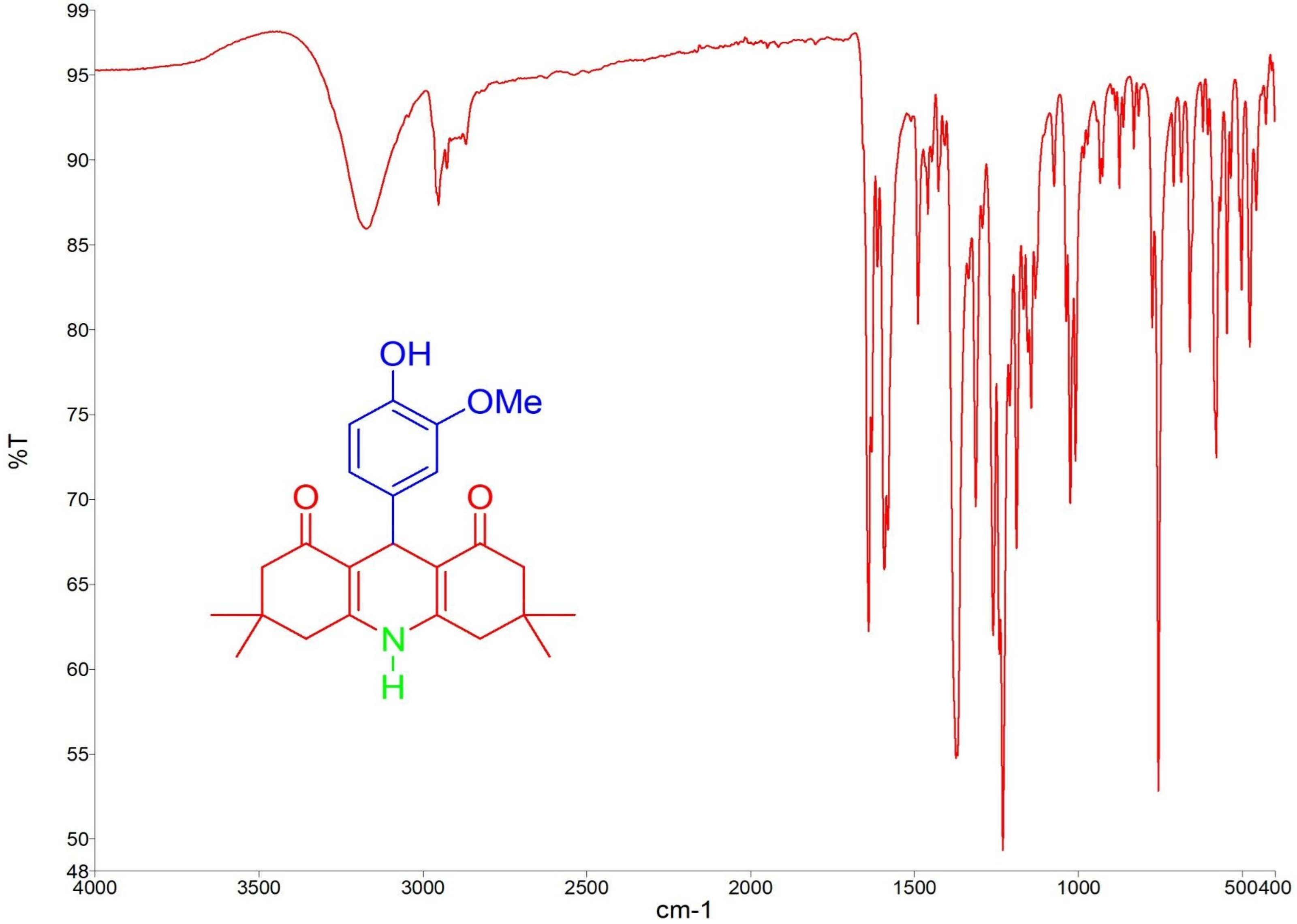

Figure S19. IR spectrum of 9-(4-hydroxy-3-methoxyphenyl)-3,3,6,6-tetramethyl-3,4,6,7,9,10-hexahydroacridine-1,8(2H,5 H)-dione (3k).

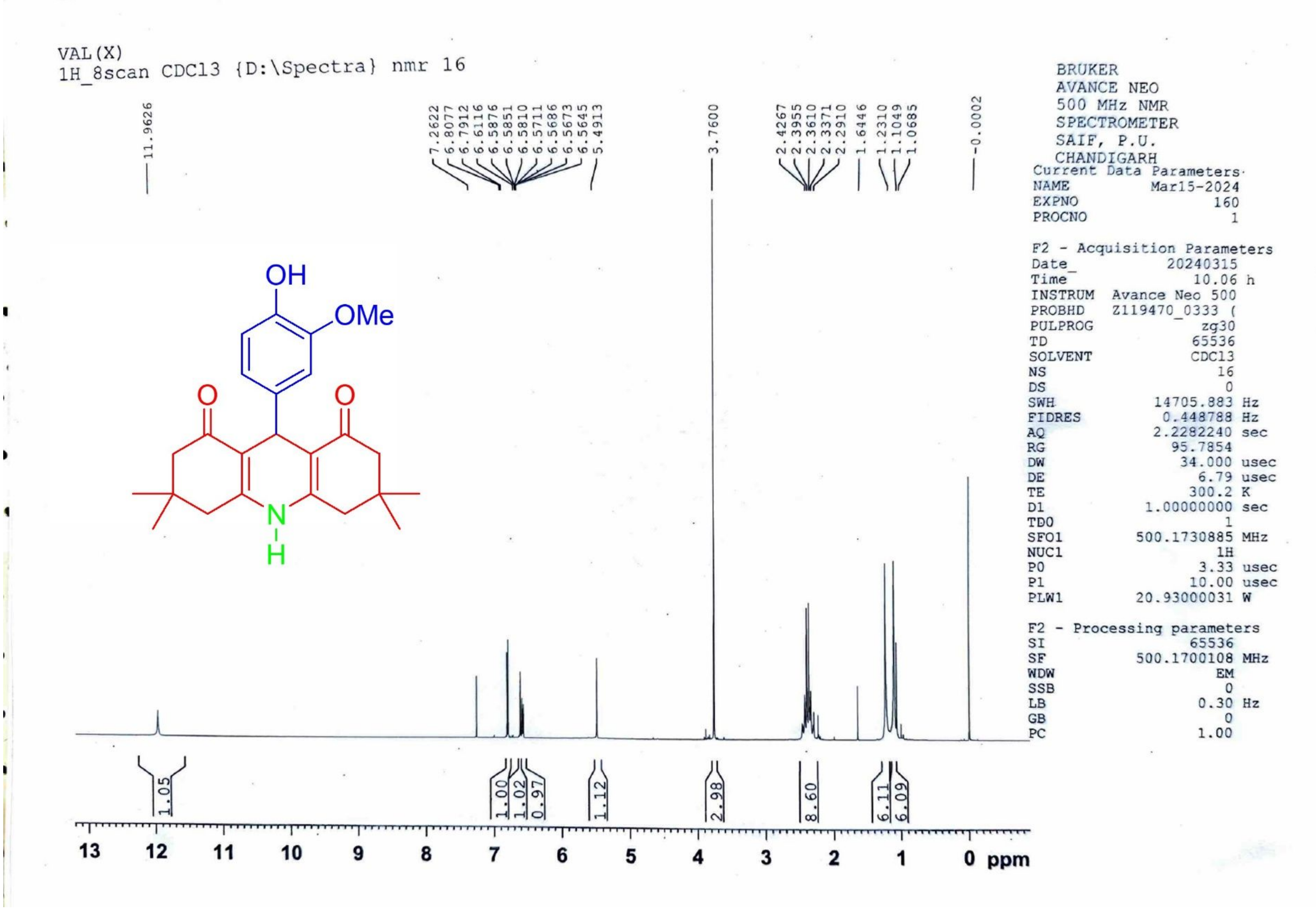

Figure S20. ^{1}H **NMR spectrum of 9-(4-hydroxy-3-methoxyphenyl)-3,3,6,6-tetramethyl-3,4,6,7,9,10-hexahydroacridine-1,8(2H,5 H)-dione (3k).**

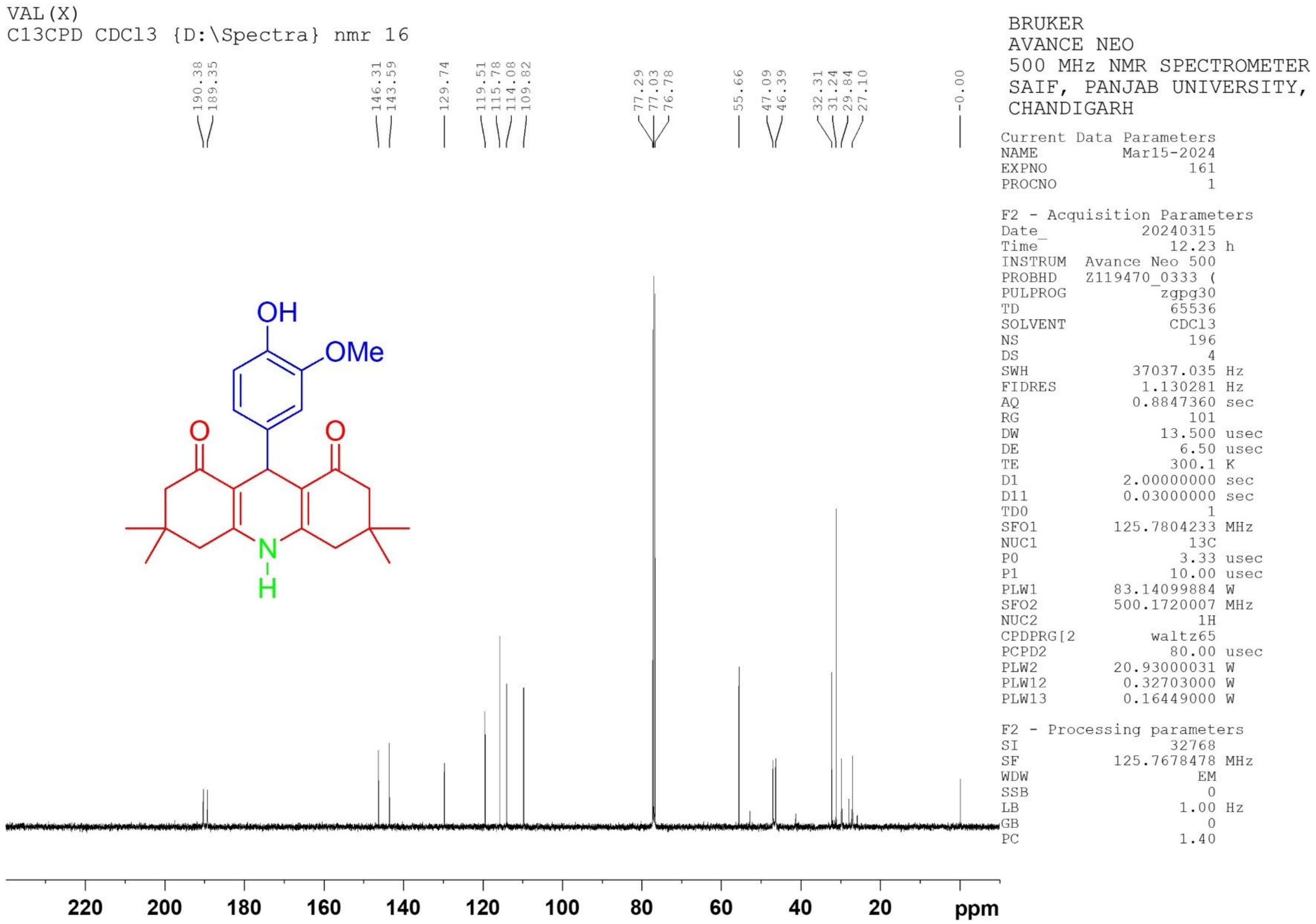

Figure S21. **^{13}C NMR spectrum of 9-(4-hydroxy-3-methoxyphenyl)-3,3,6,6-tetramethyl-3,4,6,7,9,10-hexahydroacridine-1,8(2H,5 H)-dione (3k).**

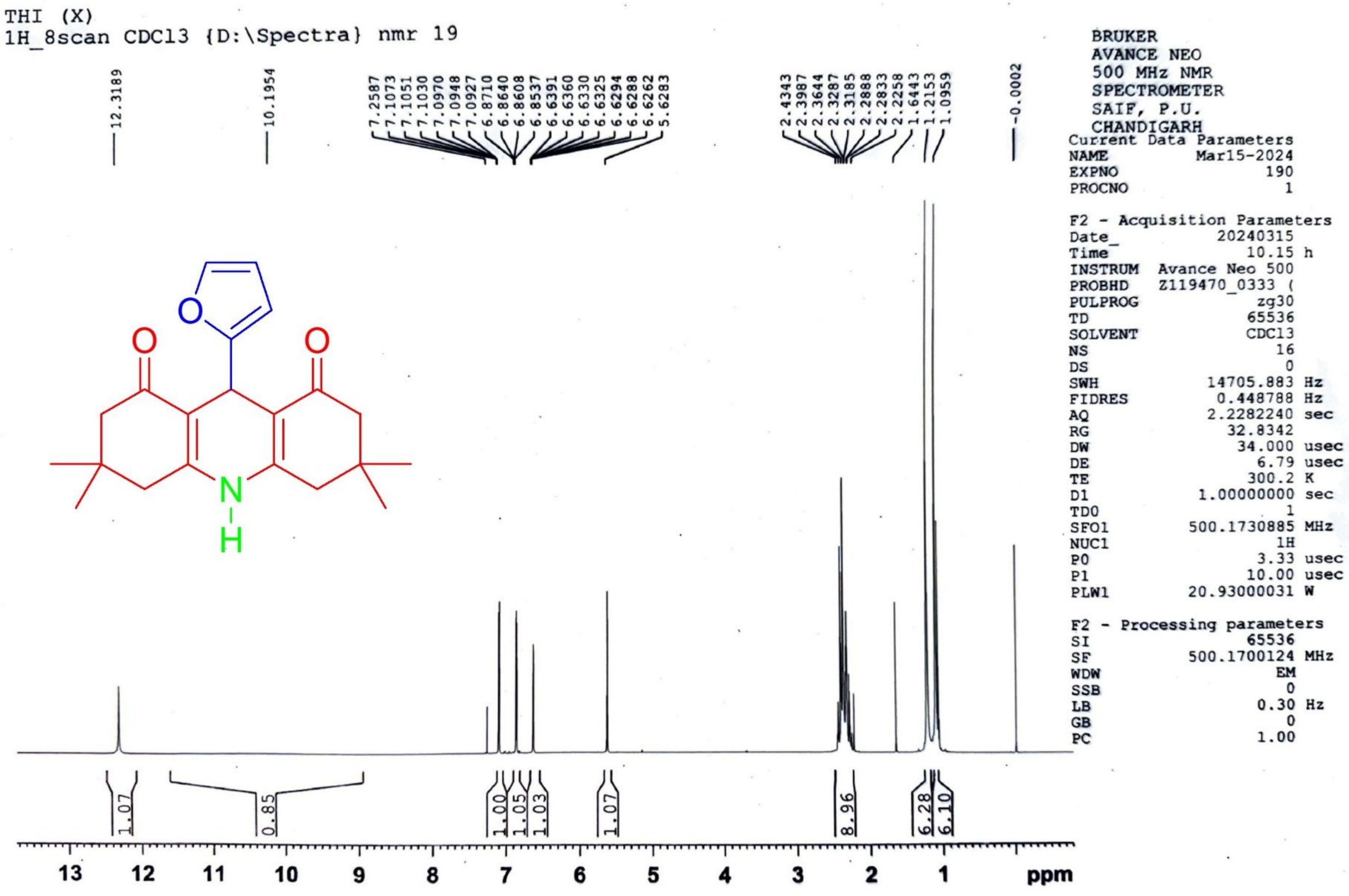

Figure S22. ^{1}H NMR spectrum of 9-(furan-2-yl)-3,3,6,6-tetramethyl-3,4,6,7-tetrahydroacridine-1,8(2H,5H,9H,10H)-dione (3l).

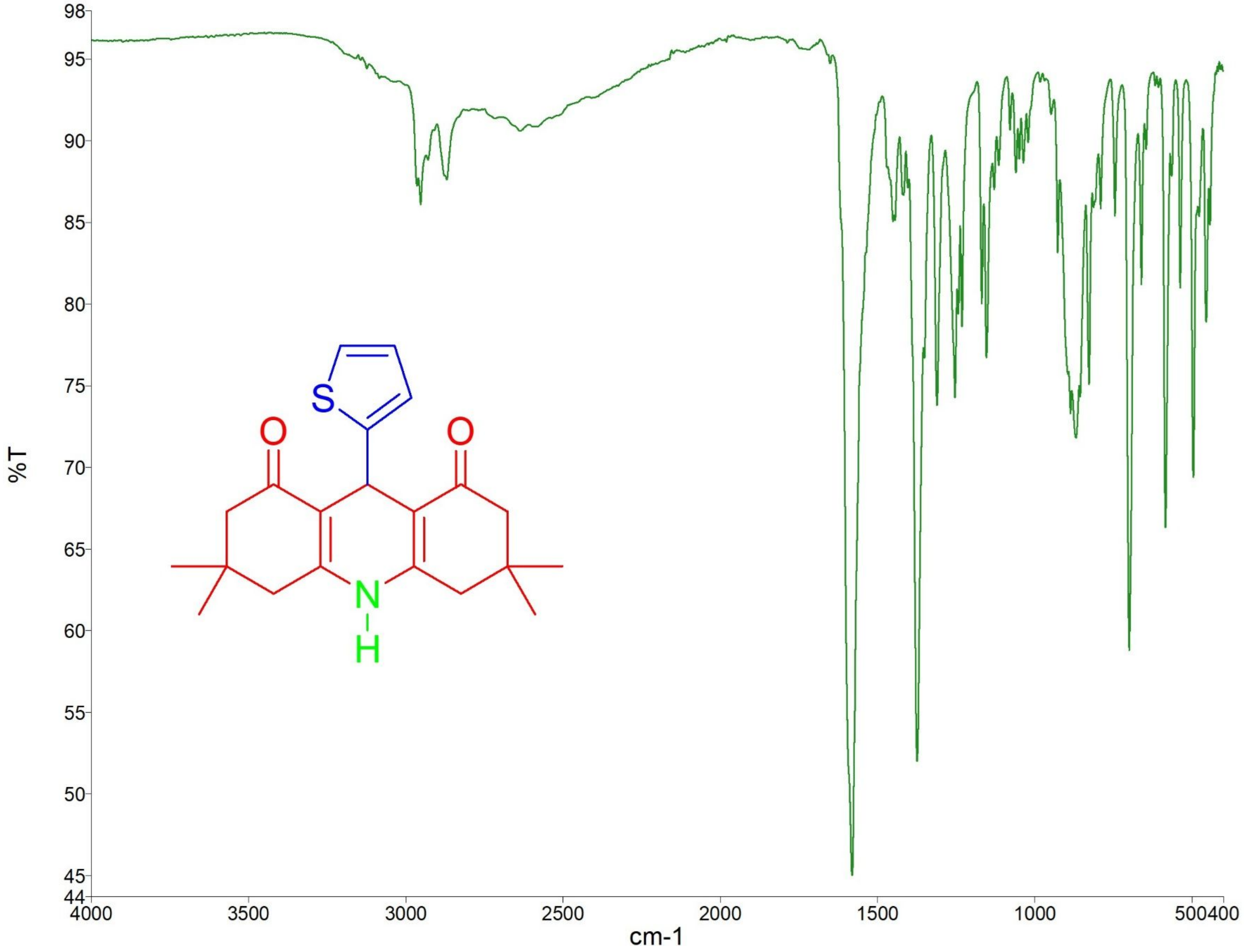

Figure S23. IR spectrum of 3,3,6,6-tetramethyl-9-(thiophen-2-yl)-3,4,6,7-tetrahydroacridine-1,8(2H,5H,9H,10H)-dione (3m).

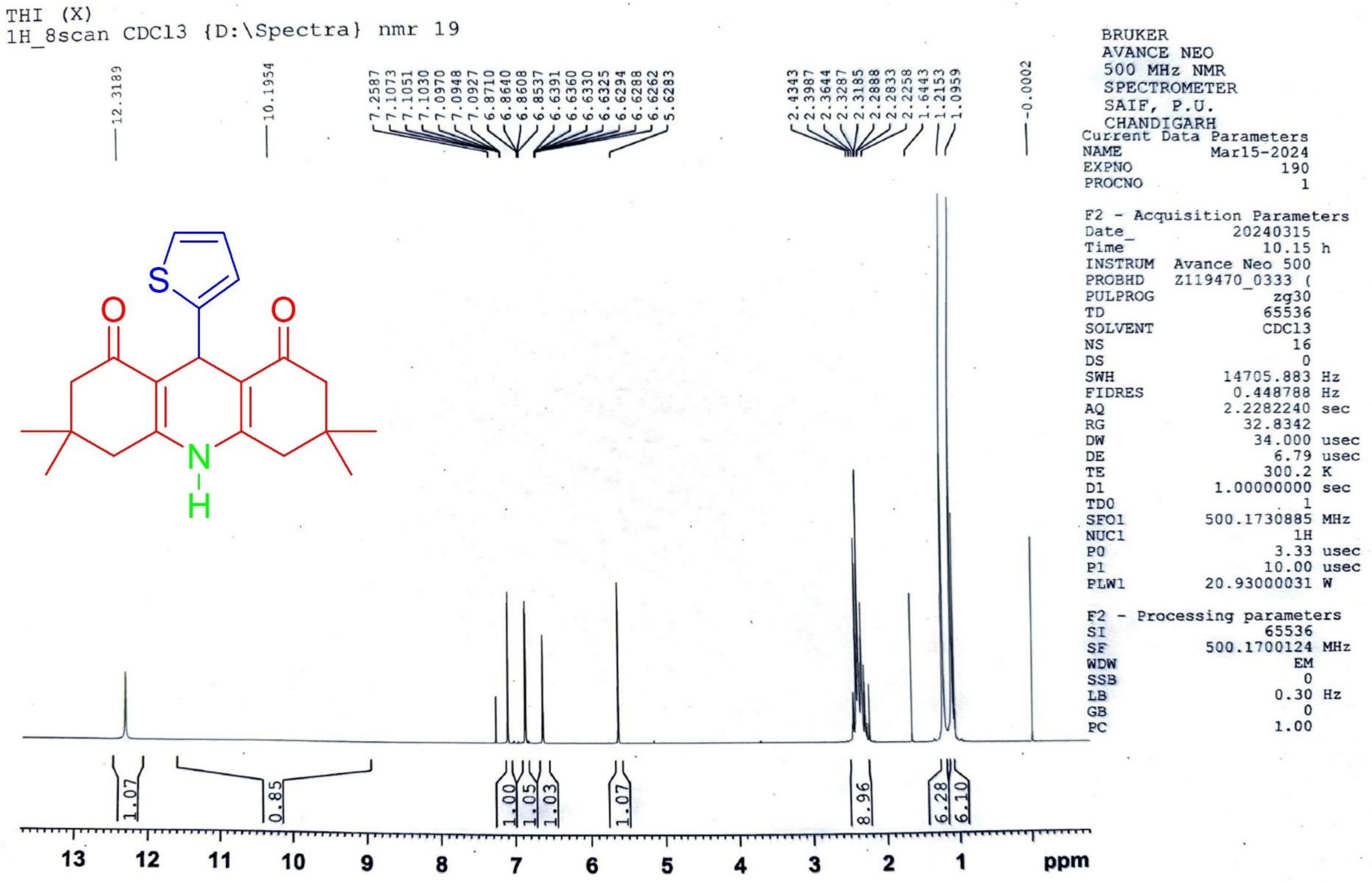

Figure S24. ^{1}H **NMR spectrum of 3,3,6,6-tetramethyl-9-(thiophen-2-yl)-3,4,6,7-tetrahydroacridine-1,8(2H,5H,9H,10H)-dione (3m).**

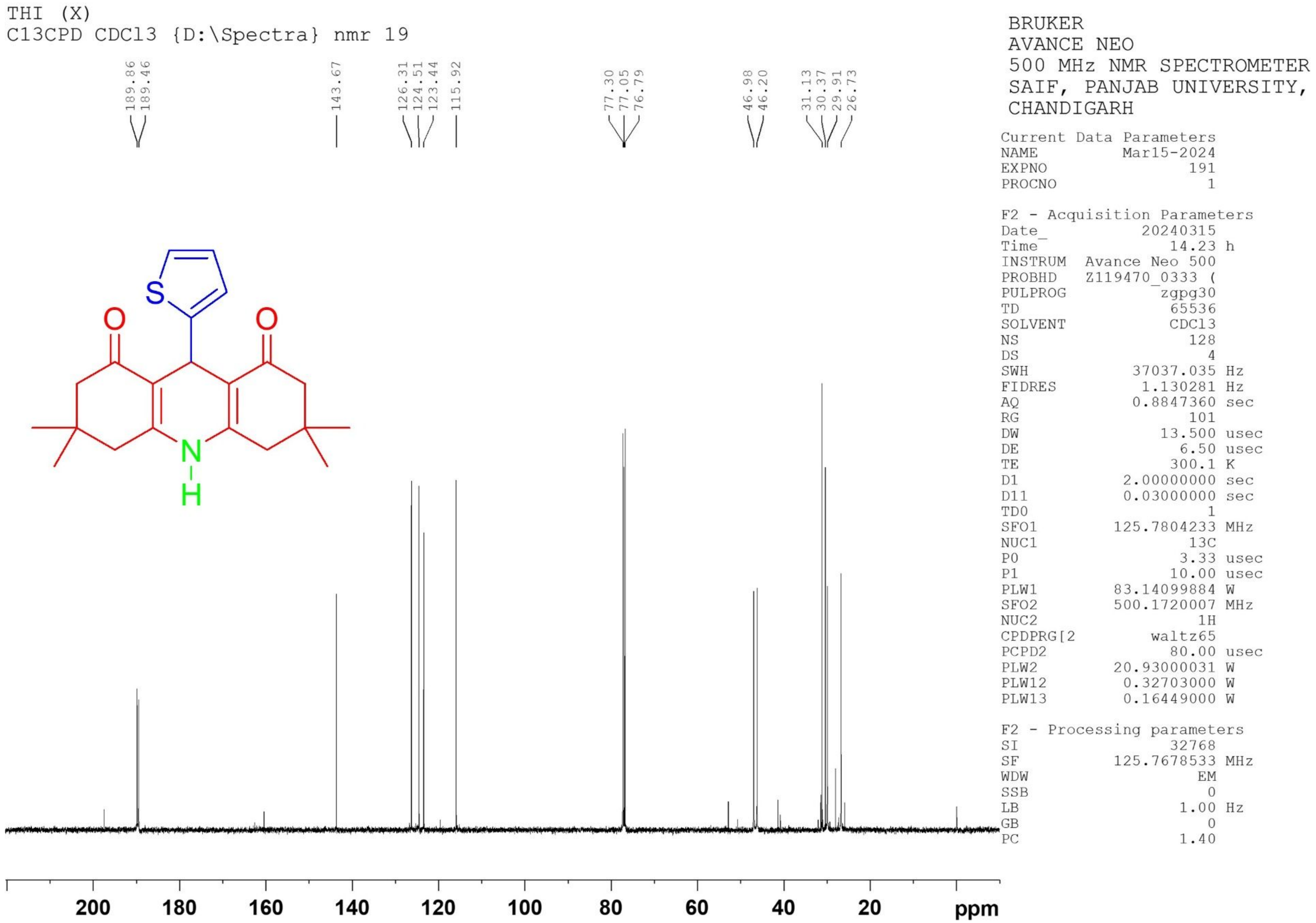

Figure S25. ^{13}**C NMR spectrum of 3,3,6,6-tetramethyl-9-(thiophen-2-yl)-3,4,6,7-tetrahydroacridine-1,8(2H,5H,9H,10H)-dione (3m).**

SI3. Reusability tests

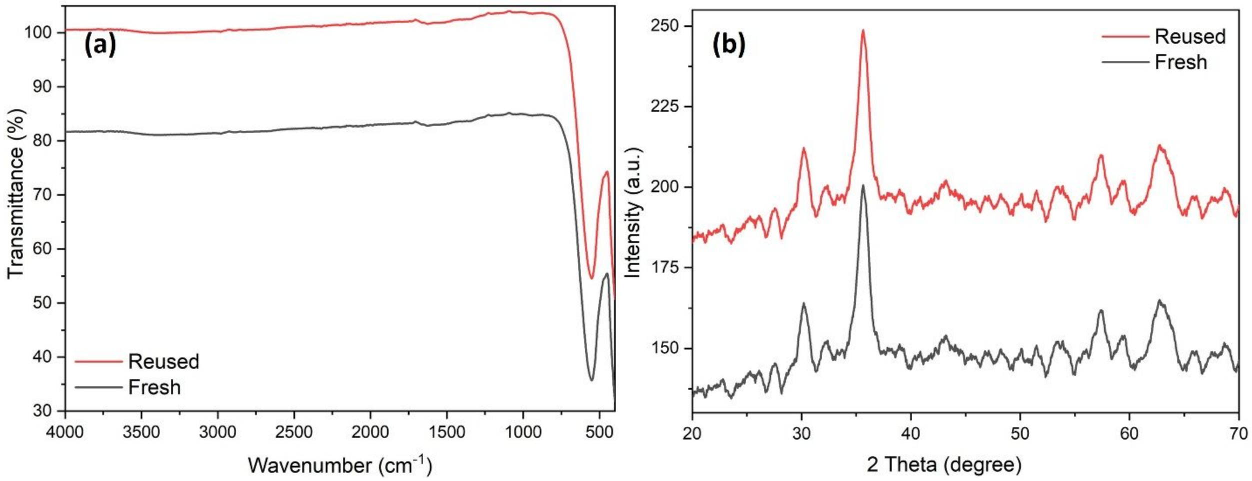

Figure S14. (a) FT-IR spectra and **(b)** XRD patterns of the Ni@Fe_3O_4 nanocatalyst before use (fresh) and after the fifth catalytic cycle.
